# Environmental Effects on Compulsive Tail Chasing in Dogs

**DOI:** 10.1371/journal.pone.0041684

**Published:** 2012-07-26

**Authors:** Katriina Tiira, Osmo Hakosalo, Lauri Kareinen, Anne Thomas, Anna Hielm-Björkman, Catherine Escriou, Paul Arnold, Hannes Lohi

**Affiliations:** 1 Research Programs Unit, Molecular Medicine, Department of Veterinary Biosciences, University of Helsinki, Helsinki, Finland; 2 The Folkhälsan Research Center, Helsinki, Finland; 3 Antagene, Animal Genetics Laboratory, Lyon, France; 4 Department of Equine and Small Animal Medicine, University of Helsinki, Helsinki, Finland; 5 National Veterinary School of Lyon, Lyon, France; 6 Program in Genetics and Genomic Biology, Hospital for Sick Children, Toronto, Ontario, Canada; Institute of Psychiatry at the Federal University of Rio de Janeiro, Brazil

## Abstract

Obsessive Compulsive Disorder (OCD) is a neuropsychiatric disorder observed both in humans and animals. Examples of Canine Compulsive Disorder (CD) include excessive tail chasing (TC), light/shadow chasing and flank sucking. We performed a questionnaire survey to investigate the characteristics of compulsive (TC) and its possible associations with environmental correlates and personality in a pet population of 368 dogs from four dog breeds. We observed an early onset of TC at 3–6 months of age and a large variation in TC frequency in all breeds, with an overrepresentation of milder cases. Almost half of the TC dogs showed lowered responsiveness during bouts and displayed also other types of compulsions more often than the controls. Interestingly, dogs that received dietary supplements, especially vitamins and minerals, expressed less TC compared to dogs that did not receive any supplements. Neutered females had less TC, suggesting an influence of ovarian hormones on TC. Tail chasers were shyer and had separated earlier from their mothers than the controls. Finally, our genetic study did not find an association between TC and CDH2, a locus previously associated with the canine flank sucking compulsion. In conclusion, the early-onset and the variable nature of the repetitive behaviour, which is affected by environmental factors such as micronutrients, neutering and maternal care, share several similar components between canine and human compulsions and supports canine TC as a model for human OCD.

## Introduction

Obsessive compulsive disorder (OCD) in humans is characterized by recurrent intrusive thoughts (obsessions) and mental rituals and repetitive behaviours (compulsions), such as ordering, cleaning or checking, which interfere with daily functioning and/or are highly distressing [1]. Between 1 and 3% of the human population worldwide suffer from OCD; a disease which often follows a chronic course and has been listed by the World Health Organization (WHO) as a leading cause of disability [2], [3]. First-line treatment of OCD in humans includes cognitive-behavioural therapy and serotonergic medication [4]. Human OCD has been linked to the serotonin and dopamine neurotransmitter systems and altered glutamate neurotransmission [5]. A strong genetic predisposition has been suggested [3], [6], [7], particularly for OCD beginning in childhood. Heritability estimates for obsessive-compulsive symptoms based on twin studies are 0.25–0.45 for adults and 0.45–0.65 for children [3], [8].

Exaggerated, inappropriate and repetitive behaviours in animals are referred to as stereotypic or compulsive behaviours. These are often compared to symptoms of human OCD, although the existence of obsessive thoughts in animals remains controversial [9]. Stereotypic behaviour in animals (observed only in captive and/or domesticated animals) are suggested to be exaggerated forms of natural behaviours, such as feeding, locomotion or predation [10], [11]. The potential causes of animal stereotypic behaviour include both genetic and environmental factors [6], [12]–[14]. For example, a genetic predisposition for OCD-like behaviours has been suggested in dogs [12], and naturally occurring compulsive behaviours have been described in rodents [11], [15]. Animal stereotypic behaviour has also been proposed to represent a coping strategy for environmentally induced stress and anxiety [16].

Spontaneous compulsive behaviours occur in many dog breeds and can take several forms including repetitive pacing, tail chasing, sucking (i.e.fabric or flank sucking), licking, chasing “invisible flies” or shadows/lights, freezing, and staring [12], [13]. The literature on canine stereotypic behaviours is mainly limited to clinical case studies based on severely affected patients in need of veterinary consultation [17]. Compulsive behaviours in dogs share clinical similarities with human OCD. Similarities between canine compulsive behaviours and their human analogues include repetitive nature, early-onset and response to medication such as serotonin reuptake inhibitors (SSRIs). Neural regions expressed in human OCD include the orbitofrontal cortex, the dorsolateral prefrontal cortex, the anterior cingulate circuit, the basal ganglia and the thalamus [18] and also the amygdala [19]. A recent study reported significantly lower 5-HT2A receptor binding indices in the frontal and temporal cortices in compulsive dogs, and also abnormal dopamine transporter rations in the left and right striatum were observed [20]. This suggests neurobiological similarity between canine and human compulsive behaviour. Consequently, canine compulsive behaviours have been suggested as a promising model for human OCD with a good face and predictive validity [12], [21]. Furthermore, a locus for the flank sucking behaviour, a compulsive behavioural disorder most often seen in Doberman Pinchers, was recently mapped to the gene cadherin 2 (CDH2) in a genome-wide association study [22]. CDH2 has also been associated with human autism [23]. Although an independent replication of this genetic finding is required, it provides further support that overlapping etiologies may lead to compulsive behaviours across species.

Tail chasing (TC) is a classic compulsive behaviour in dogs. A variant of TC is “spinning”, in which the affected dog spins rapidly in tight circles without apparent interest in the tail. TC often occurs in bouts and might include episodes in which the dog stares at its tail quietly for a while before resuming chasing. TC is suggested to have a genetic predisposition as it is more common in certain breeds, such as Bull Terriers, German Shepherds [13] and Staffordshire Bull Terriers [24]. TC was reported to occur in Bull Terriers together with extreme aggression and fear, presenting as a syndrome suggested to resemble partial seizures, as these dogs had abnormal EEG results and partial responses to phenobarbital medication [25]. A recent clinical and questionnaire study of over 300 TC Bull Terriers showed a higher prevalence in males and found an association of TC with trance- like -behavior and episodic aggression [26]. The authors also suggest that TC in Bull Terriers might present with autistic features.

Our aim was to study the phenotypic and genetic characteristics of compulsive TC, including possible environmental and personality correlates. Towards these aims, we have developed detailed behavioural questionnaires for dog owners and collected data from a population sample of 368 dogs from four different breeds, including Bull Terriers (Standard BT and Miniature MBT), Staffordshire Bull Terriers (SBT) and German Shepherds (GS). Furthermore, DNA samples were collected from 181 dogs and a candidate gene approach was used to test the association of TC with the CDH2 locus.

## Results

### Characteristics of Tail Chasing Behaviour

**Figure 1:**
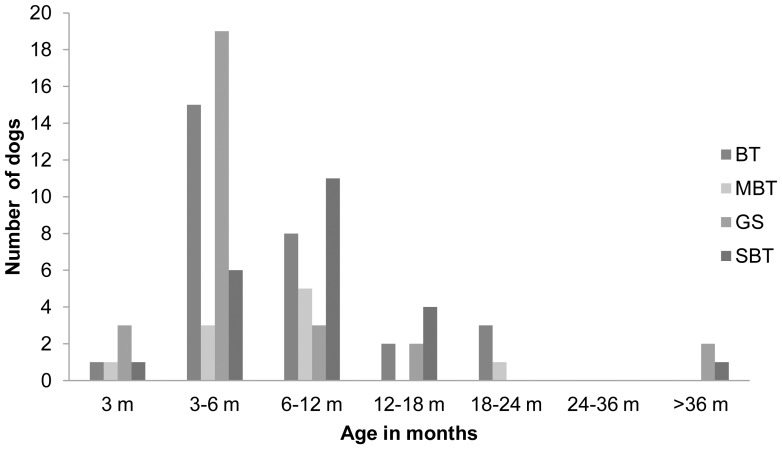
The age of onset of tail chasing (TC) behaviour in each breed. BT =  Bull Terrier, standard, MBT  =  Bull Terrier, miniature, GS =  German Shepherd, SBT  =  Staffordshire Bull Terrier.

Our questionnaire (**Attachment S1**) yielded data for a total of 368 dogs from four breeds. This dataset indicated the typical onset of TC at 3–6 months of age in BT and GS and 6–24 months in MBT and SBT (**Fig. 1**). Three dogs were reported with the age of onset >3 years (one SBT and one GS were bought at the age of 2 years and one GS started TC after the arrival of another male dog into the same household). Short video clips of TC behaviour of BT, GS and SBT cases are included in the **Movie S1, S2 and S3**.

**Figure 2:**
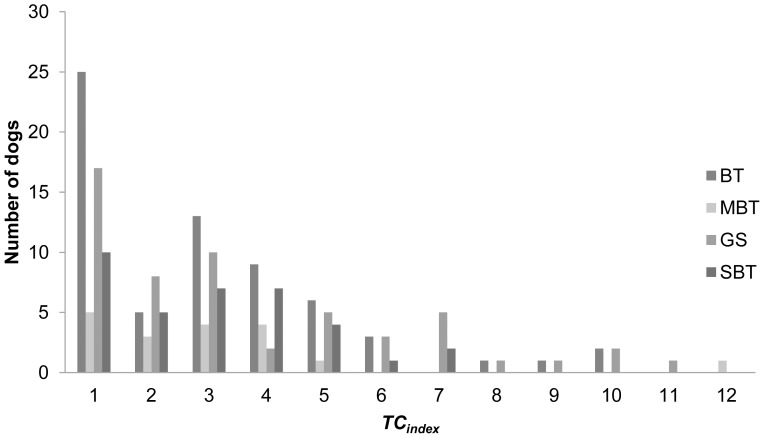
Distribution of the dogs according to severity of tail chasing behavior. Only individuals that exhibit tail chasing behavior are included. Severity is described by the *TC_index_*, which is a sum of two questions “Does your dog chase its tail?”(scale 0–6) and “On average, how much time on a normal day does the dog spent in chasing its tail? “(scale 0–6). (BT =  Bull Terrier, standard, MBT  =  Bull Terrier, miniature, GS =  German Shepherd, SBT  = Staffordshire Bull Terrier).

The frequency of TC behaviour showed a large variation in our study populations. Some of the dogs had only few episodes during their lifetime, while the most severe cases were engaged in TC several times a day (**Fig. 2**). Our study included 17 litters with two or more TC littermates, and we observed variation in the severity of TC also within a litter (**Figure S1**). In relation to the number of returned questionnaires per breed, the least TC (*TC_index_ = 1*–*12)* dogs were reported in GS (54/180), and the most in BT (83/122) (Kruskal-Wallis Test *χ^2^*_2,366_ = 35.1, p<0.001). GS had also lower mean *TC_index_* compared with SBT and BT (Kruskal-Wallis Test *χ^2^*_2,367_ = 45.1, p<0.001). These results may reflect the actual breed differences in tail chasing, but may also be the result of a difference in sampling methods between breeds, and activity of breed clubs in sharing information from the study to the dog owners.

There were no differences in TC prevalence between males and females, although there were slightly more females in the control group (Chi-square test *χ^2^*_1,367_ = 2.92, p = 0.095). Males and females did not differ in the severity defined by the *TC_index_*, however, we found that neutered individuals had lower *TC_index_* compared to non-neutered individuals in the pooled data (p = 0.008, **Table 1**). Although females and males did not seem to differ in their relationship with neutering and TC, when including only individuals with *TC_index_* = 0 and *TC_index_* = **2**–12 (leaving out dogs with *TC_index_ = 1)*, the effect of the neutering was significant only among females (all breeds pooled, Chi-square test: females *χ^2^*_1,153_ = 4.46, p = 0.035; males *χ^2^*_1,151_ = 0.056, p = *ns*), suggesting that neutering in females may have a controlling effect on TC behaviour. The total number of neutered females in our study was 60, whereas there were only 18 sterilized, males preventing us from drawing further conclusions on the effect of sterilization on TC in males. A within-breed analysis of the effect of neutering on TC showed a significant association only in SBT (**Table 1**). As we also had information on the neutering age of 42 females (all breeds pooled), we further investigated whether the neutering age affected TC. The age of neutering did not associate with *TC_index_* (all breeds n = 42, r = 0.047, p = ns; SBT n = 18, r = 0.033, p = ns), nor were there any differences in TC between dogs neutered at younger age (<2 yr, n = 10) and dogs neutered at an older age (>4 yrs, n = 10) (*χ^2^*_1,20_ = 0.66, p = ns, all breeds).

**Table 1:** Association of tail chasing behaviour^a^ and environmental factors based on pooled data (N = 368) and for each breed separately.

*Source – pooled data (nb)* b *(N = 308)*	*Num* c *df*	*Den* d *df*	*F value*	P^e^
*dietary nutrients (yes/no)*	1	302	11.56	<0.001
*neutered (yes/no)*	1	302	7.03	0.008
*Breed*	2	302	11.86	0.003
*number of dogs at home*	1	302	10.15	0.001
***Source – Bull Terriers (nb)*** b ***(N = 109)***	***Num df***	***Den df***	***Chi-square***	**P**
*dietary nutrients (yes/no)*	1	105	8.71	0.003
*number of dogs at home*	1	105	9.44	0.002
*amount of socialization*	1	105	3.01	0.083
***Source – Staffordshire Bull Terriers (nb)*** b ***(N = 58)***	***Num df***	***Den df***	***Chi-square***	**P**
*Neutered*	1	54	9.43	0.002
gender* neutered	1	54	6.14	0.013
*number of children*	1	54	14.08	<0.001
***Source – German Shepherd)(pd)*** f ***(N = 178)***	***Num df***	***Den df***	***F value***	**P**
***dietary nutrients (yes/no)***	1	176	3.75	0.053

a *TC_index_* (*TC_index_* = 0–12) is used as a response variable.

b nb = negative binomial distribution.

c Num = numerator.

d Den =  denominator.

e Only the variables with P values <0.1 are presented for each model. Type 3 analysis statistics are presented (type III analysis does not depend in the order in which the terms are presented in the model).

f pd = Poisson distribution, dscaled.

**Figure 3:**
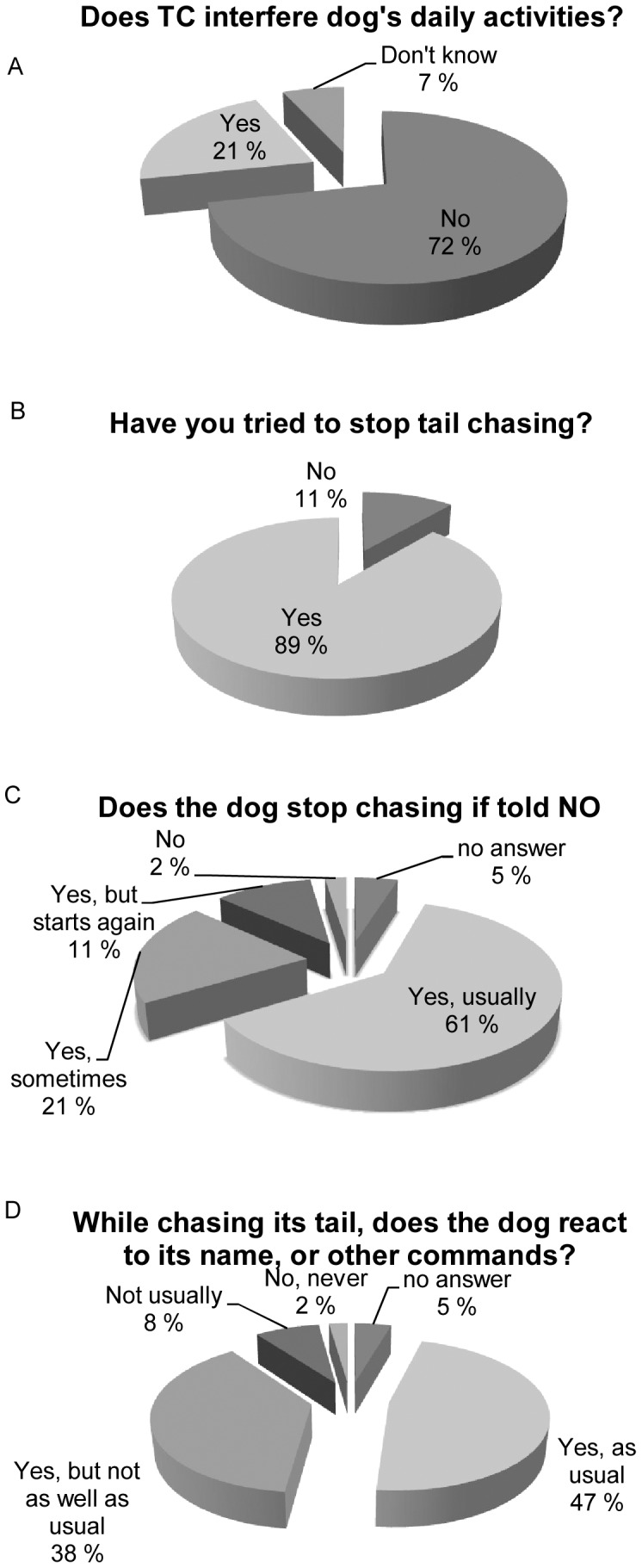
Summary of the owners’ perceptions on their dog’s tail chasing (TC) behaviour. Summary of the answers to the following questions a) does TC interfere with dog’s daily activities?, b) has the owners tried to stop the behaviour, c) does the dog stop tail chasing when it is told to and d) while chasing its tail, does the dog react to its name, or other commands? Graphs represent a pooled data analysis including all breeds.

Although most owners (72%) reported that TC did not affect the dogs’ daily activities, the majority of them had tried to stop the behaviour (89%, **Fig. 3a and b**). Altogether 61% of the owners were able to stop their dogs’ TC, while approximately one third (34%) reported difficulties in stopping the behaviour (**Fig. 3c**). Almost half of the TC dogs (47%) responded normally to owners’ calls during the TC, while the other half (48%) showed lowered responsiveness (**Fig. 3d**). The breeds differed in responsiveness: BT were reported more often to have lowered responsiveness, compared to SBT and GS *x^2^_2,_*_88_ = 14.8, p<0.001 (analysis including only tail chasers *TC_index_>2)*. Differences in general responsiveness may exist between breeds regardless of TC, however, we do not have the data to investigate the general responsiveness among the controls. The more the dog chased its tail, the more difficult it was for the owner to stop the behaviour (Spearman correlation coefficient; *N* = 83, *r* = 0.38, p<0.001) and the less it reacted to the owners’ calls (Spearman correlation coefficient; *N* = 89, *r* = 0.27, p = 0.009), indicating that the severity of the TC affects the dog’s awareness. Only one dog in our study (MBT, male) was reported to be on medication for TC (fluoxetine). The medication had markedly reduced TC, but the dog still suffers from occasional hallucinatory fly-catching compulsions.

**Figure 4:**
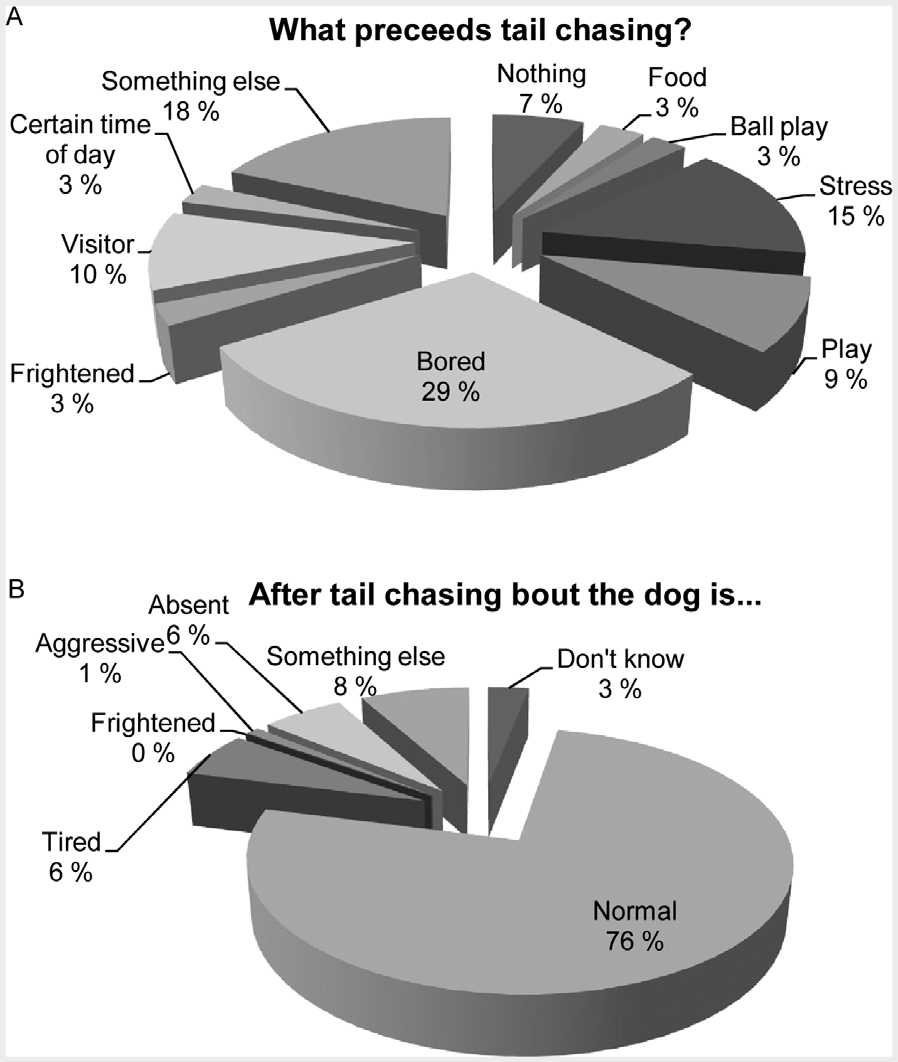
Summary of the owners’ descriptions of the tail chasing (TC) situations. (a) what precedes tail chasing? (b) how does your dog look after tail chasing? Graphs represent a pooled data analysis including all breeds.

We asked owners to describe the circumstances preceding the episodes in order to investigate whether there were any particular signs or behaviours which might precede or trigger TC. TC was most often attributed to the dog’s boredom or lack of activities (29%), stressful events (15%) or “something else” (18%) (**Fig. 4a**). The category “something else” was explained in more detail in 17 answers; most frequently TC was associated with the dog’s excitement or happiness (5/17), with food (2/17) or with frustration (2/17). Only 7% of the owners could not define particular conditions that preceded the TC. Most owners (76%) reported their dogs to have been in normal mental and physical states after the TC (**Fig. 4b**). Breed differences were observed in preceding circumstances only in the case of stressful events: GS owners reported more stress-linked TC compared to the other breeds (Kruskal-Wallis Test *x^2^_2,_*_89_ = 6.77, p = 0.034). In addition, we compared the demographic info, dog phenotype and dog personality of dogs whose owners had replied boredom or stress as triggers and those whose owners indicated some other trigger-option. ‘Bored’ dogs chased their tails significantly more (Kruskal-Wallis Test *x^2^_1,164_* = 21.48, p<0.001) than dogs, whose owners had indicated some other option as the trigger for TC. The same applied for the ‘stressed’ dogs; they had also a significantly higher *TC_index_* compared to the other trigger-options (*x^2^_1,164_* = 23.44, p<0.001). ‘Bored’ dogs had also experienced better maternal care (*x^2^_1,37_* = 5.86, p = 0.016). ‘Stressed’ dogs were given less dietary nutrients (Fishers’ exact test, p* = *0.042) and their TC tended to start at a later age (*x^2^_1,91_* = 3.38, p = 0.066). No other differences were observed between the dogs that had different triggers for TC.

**Figure 5:**
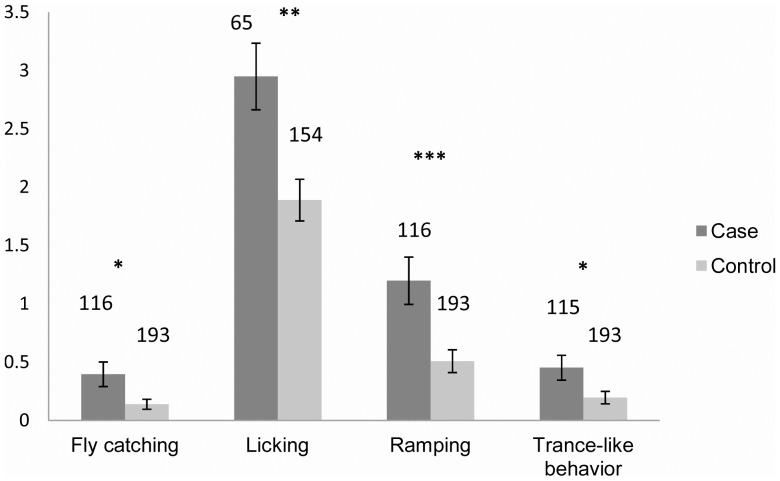
The presence of other compulsions such as fly catching/light chasing behaviour, licking, repetitive pacing and freezing (trance-like behaviour) in case and control dogs. Case dogs have *TC_index_*≥2, whereas control dogs have never chased tail tails *(TC_index_ = 0*). As a measure of the compulsive behaviours (represented in the Y axis) we use index calculated as the sum of numerical answers to questions “Does your dog freeze/chase lights etc.?” from ‘never’ to ‘multiple times per day’ and “On average, how much time on a normal day does the dog spent in that specific behaviour?” from 0 min up to 5 hours or more (**Attachment S1**). All breeds are pooled for the analysis. Sample size for each group is marked on the top of each bar. Statistical significance is marked following *<0.05, **<0.01, ***<0.001.

In a pooled breed analysis we found tail chasers to also have other compulsions more often than the controls, such as ‘invisible flies’ or light-chasing behaviours (Kruskal-Wallis, *x^2^_1,_*_308_ = 4.42, p = 0.035), licking (*x^2^* = 10.37, p = 0.001), repetitive pacing (*x^2^_1,_*_308_ = 11.23, p<0.001) and freezing or trance-like behaviour (*x^2^_1,_*_308_ = 5.08, p = 0.024) (**Fig. 5**). Repetitive pacing refers to excessive pacing, where the dog is either running or walking in a repetitive pattern. The pattern may be a circle, a number eight or something else. In the case of licking and repetitive pacing, the owners reported only the time spend for that specific activity for each dog, and therefore most of these individuals with rather high frequencies cannot be directly categorized as being compulsive lickers or pacers. Nevertheless, this measure serves as an indication of increased activity in potentially compulsive behaviours. Freezing was mostly seen in BT (BTs 40, MBT3), but also six SBTs and two GSs were reported to exhibit this behaviour.

### Environmental Factors

The possible association of environmental factors with TC was investigated using two different models: one including all individuals (n = 368) and the *TC_index_* as a response variable, and the other including only the most severe tail chasers (*TC_index_* 5–12, n = 35) and the controls (*TC_index_ = *0, n = 150). The latter comparison was included to investigate whether the most severe tail chasers were also affected by environmental factors. We found that gender, amount of exercise, amount of activities, time spent alone, age of arrival to the household, number of adults in the household, number of diagnosed diseases, type of food, or birth place were not associated with TC in any of the analyses in a pooled sample or a breed-specific analysis (data not shown), and these same factors were dropped from the final models (data not shown).

Although we did not find an association to food types, we did observe a statistically significant association of TC with dietary nutrients (p<0.001) (**Table 1**). Those dogs that received (sometimes or regularly) dietary nutrients had less TC compared to the dogs not receiving any. The interaction term breed x dietary nutrients was not significant, indicating that the direction of association was similar in each breed, although the within-breed analysis revealed statistical significance only in BT (p = 0.003) and a non-significant tendency in GS (p = 0.053, **Table 1**). The effect of dietary nutrients was also observed in the analysis including *only* tail chasers: dogs that had received dietary nutrients had less TC compared to dogs receiving no dietary nutrients (**Table 2**). This was especially the case in BT and SBT, but not in GS (**Table 2**).

**Table 2:** Association of tail chasing behaviour and dietary nutrients *only* among tail chasers (*TC_index_≥2*)^a^.

*Source – pooled data (N = 116)*	*df*	*Chi-square*	P
*TC_index_*	1	8.73	0.003
*Breed*	2	11.90	0.003
*Breed* * *TC_index_*	2	8.32	0.016
***Source – Bull Terriers (N = 53)***	***df***	***Chi-square***	**P**
*TC_index_*	1	7.24	0.007
***Source – Staffordshire Bull Terriers (N = 27)***	***df***	***Chi-square***	**P**
*TC_index_*	1	8.01	0.005
***Source – German Shepherd (N = 36)***	***df***	***Chi-square***	**P**
***TC_index_***	1	0.20	0.657

a Binary variable *given nutrients/not given nutrients* was used as a response variable in the model. Sex and sex.

* *TC_index_* were included in the preliminary analysis, but were nonsignificant and were dropped out. Results for both pooled and breed-wise analyses are presented. Type 3 analysis statistics are presented.

**Figure 6:**
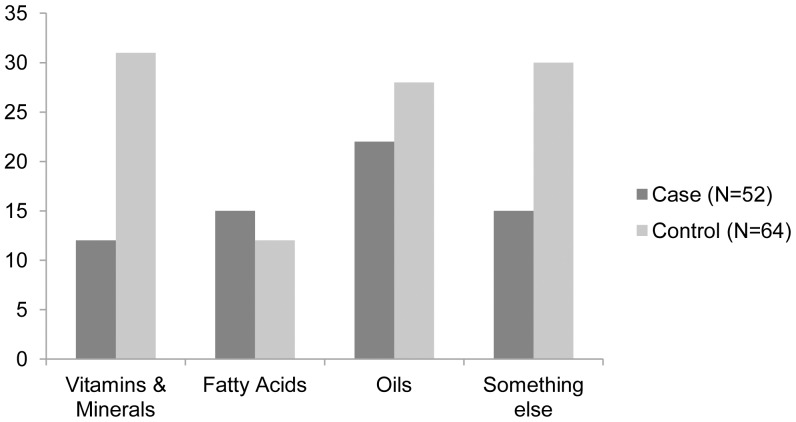
Number of case and control dogs who received dietary supplements. Case dogs have *TC_index_*≥2 whereas control dogs have never chased tail tails *(TC_index_ = 0* ). Dietary supplements are divided into the following categories: vitamins, minerals, Omega-3 and -6 fatty acids, and “something else”. All breeds are pooled for the analysis (n = 116). Statistical significance is marked as following *<0.05, **<0.01, ***<0.001.

An analysis of the specific diet supplement groups (**Fig. 6**) revealed that compared to the controls, significantly fewer tail chasers (*TC_index_*≥2, all breeds pooled N = 116) were receiving vitamins (Fishers’ exact test, p* = *0.02) and minerals (Fishers’ exact test, p* = *0.04). Also, individuals receiving vitamins (*x^2^_1,_*_146_ = 8.47, p = 0.004) and minerals (*x^2^_1,_*_146_ = 7.57, p = 0.006) chased their tails less than dogs that were not receiving these dietary nutrients. No difference was found between the case and control groups in receiving Omega-3, Omega-6 or a group labeled “something else” (respectively; Fishers’ exact test, p = 0.56; p = 0.70 and p = 0.46). We further focused on the micronutrients within the vitamin and mineral groups, and included only those vitamins (n = 12) and minerals (n = 10) into the analysis which had been given to more than 25 dogs. Among these micronutrients only Mg (p = 0.05), B5 (p = 0.037), B6 (p = 0.0003), B12 (p = 0.04) and vitamin-C (p = 0.04) differed between the cases and the controls, however after the Bonferroni correction for multiple testing, only vitamin B6 showed a statistical difference (p = 0.004) between the cases and the controls. Fewer tail chasers had received vitamin B6 compared to the controls. Furthermore, tail chasers that received vitamin B6 (*x^2^_1,_*_146_ = 13.96, p = 0.0002, after Bonferroni correction p = 0.001) or vitamin-C (*x^2^_1,_*_146_ = 7.78, p = 0.0002, after Bonferroni correction p = 0.027) had significantly less tail chasing compared to those tail chasers not given any vitamin B6 or vitamin C.

The number of other dogs in the household also affected TC. Dogs that lived with many conspecifics chased less compared with the dogs living alone or having fewer companions at home (p = 0.001, **Table 1**). A within-breed analysis revealed that the number of conspecifics was an important factor in BT (**Table 1**), but not in SBT or GS (data not shown). Similarly, SBTs that lived in households with more children chased their tails less than dogs living in households with fewer or no-children (**Table 1**).

Finally, we performed the same analyses described above using the pooled data (all breeds) including *only* the most severe tail chasers (*TC_index_* 5–12; tail chasing is ‘frequent’ to ‘multiple times per day’) and the controls. In this analysis, dietary supplements (*x^2^_1,185_* = 11.32, p<0.001), the amount of conspecifics at home (*x^2^_1,185_* = 9.63, p = 0.002) and sterilization (*x^2^_1,185_* = 6.05, p = 0.014) were the only TC-associated factors (generalized linear model, binomial distribution).

The effect of maternal care and age of separation (from the mother) on TC was also evaluated using a generalized linear model with a binomial distribution. Tail chasers had experienced lower quality care (*χ^2^*_1, 74_ = 5.64, *p* = 0.018, all breeds pooled) and were separated earlier from their mothers (*χ^2^*_1, 74_ = 4.40, p = 0.036) compared to dogs with no tail chasing. TC dogs had been separated from their mothers on average at 7 weeks of age, whereas in the control group the average age of separation was 8 weeks. There were no differences between females or males or between breeds (data not shown). When only the most severe cases (*TC_index_* 5–12, n = 16) were compared with the controls, the only notable difference was a trend towards decreased maternal care between severely affected TC cases and controls (n = 32) (*χ^2^*_1, 48_ = 3.17, p = 0.075).

### Tail Chaser’s Personality

The personality of the dogs was evaluated using a separate owner-filled questionnaire (**Attachment S2**) of dogs with a *TC_index_ = *0 or *TC_index_*≥2. We received a total of 129 Dog Personality questionnaires (BT standard and miniature 60, SBT 44, GS 25). Four factors were derived from the Dog Personality questionnaire using factor analysis. These factors were named Shyness/Boldness, Sociability/Dogs (sociability towards dogs), Amicability/Humans (friendliness towards humans), and Aggressiveness/Humans (aggressiveness towards humans) based on the highest loading questions in each factor (**Table 3**). Cronbach’s alpha was found to be good for each of the factors, indicating high internal consistency [27] (Shyness/Boldness 0.88, Sociability/Dogs 0.88, Amicability/Humans 0.87, Aggressiveness/Humans 0.82).

**Table 3:** Factors and loading values of each question derived from the Dog Personality Questionnaire (**Attachment S2**)^a^.

*Question*	*Shy/Boldness*	*Soc/Dogs*	*Amic/Hum*	*Aggr/Humans*
Friendliness when stroked by adult			**0.86**	
Friendliness when stroked by child			**0.66**	
Friendliness when quest at home			**0.65**	
Friendliness towards vet			**0.67**	0.35
Friendliness towards large dog		**0.87**		
Friendliness towards small dog		**0.86**		
Friendliness towards aggressive dog		**0.67**		
Fearfulness when stroked by adult	**0.79**		0.35	
Fearfulness when stroked by child	**0.73**		0.33	
Fearfulness when quest at home	**0.73**			
Aggressiveness when stroked by adult				**0.82**
Aggressiveness when stroked by child				**0.74**
Aggressiveness when quest at home				**0.68**
Aggressiveness towards vet				**0.69**
Aggressiveness towards large dog		**0.80**		
Aggressiveness towards small dog		**0.78**		
Aggressiveness towards aggr. dog		**0.72**		
Fear in dog shows, or other events	**0.47**		0.32	
Fear in shopping malls	**0.81**			
Fear in traffic	**0.70**			
Fear towards novel object	**0.57**			
Eagerness to socialize with strangers			**0.69**	0.32
Eagerness to explore novel space	**0.57**		0.48	

a Answers of all breeds were pooled together (N = 129), and *TC_index_* of the dogs varied from 0 to 2–12. If the question has loadings higher than 0.30 on several factors, question has been included in that factor for which it has the highest loading.

**Figure 7:**
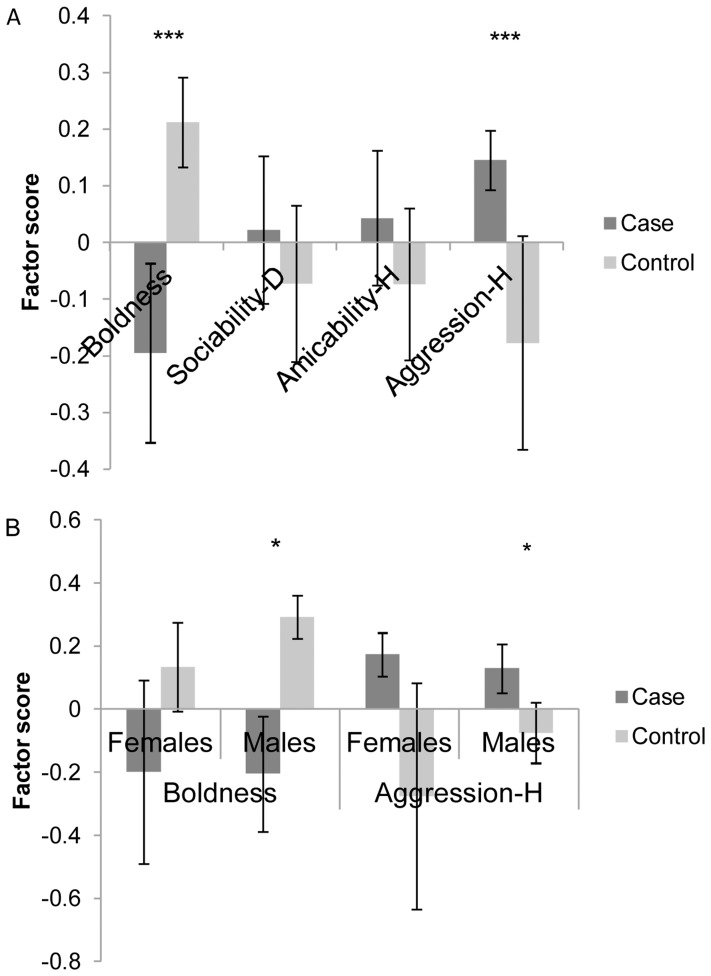
Personality factors of the case and control dogs. Case dogs have *TC_index_*≥2 whereas control dogs have never chased tail tails *(TC_index_ = 0)*. a) Factor scores (±SE) of cases and controls (all breeds pooled, n = 110) for each of the four factors labelled as Boldness (Shyness/Boldness), Sociability-D (sociability towards dogs), Amicability-H (friendliness towards humans), and Aggressiveness-H (aggressiveness towards humans). b) Factor scores (±SE) of cases and controls (all breeds pooled, n = 110) separately for females and males for two factors labelled as Boldness (Shyness/Boldness) and Aggressiveness-H (aggressiveness towards humans). Note that in the factor Aggressiveness-H, the higher the score, the less aggressive the individual is. Statistical significance is marked following *<0.05, **<0.01, ***<0.001.

The pooled analysis including all breeds indicated that tail chasers (n = 59) are generally shyer (*χ^2^*_1,110_ = 14.40, p<0.001) and less aggressive towards humans (*χ^2^*_1,110_ = 11.73, p<0.001) than non-tail chasers (n = 51) (**Fig. 7a**). Both Shyness/Boldness and Aggressiveness/Humans had a significant interaction with gender, revealing that this effect is present particularly in males. Male tail chasers (n = 34) were shyer (sex* Shyness/Boldness: *χ^2^*_2,110_ = 5.46, p = 0.0194) and less aggressive (sex* Aggressiveness/Humans: *χ^2^*_2,110_ = 5.94, p = 0.0148) compared to the control males, although females (n = 25) also had the same non-significant trend (**Fig. 7b**). We did not find differences in the Sociability/Dogs (*χ^2^*_1,110_ = 0.17, p = ns) or Amicability/Humans (*χ^2^*_1,110_ = 0.03, p = ns) personality factors between tail chasers and non-chasers. When breeds were analysed separately, we found statistically significant personality differences only in BT, where tail chasers (n = 31) were shyer (*χ^2^*_1, 50_ = 10.31, p = 0.001) compared to the controls (n = 19), and the male dogs were less aggressive towards humans (sex* Aggressiveness/Humans: *χ^2^*_2,50_ = 5.56, p = 0.038) than the female dogs.

We analysed questions that were left out of the factor analysis (Q7, 6d) due to low communality values and (Q8) binomial distribution, separately. Pooled analysis of the breeds showed differences between the breeds in these questions and we therefore analysed each breed separately. Both BT and GS tail chasers had more noise phobia (Q7) (BT *χ^2^*_1, 52_ = 6.02, p = 0.014; GS *χ^2^*_1, 25_ = 4.23, p = 0.040) compared to the controls and the same non-significant trend was also observed in SBT (fireworks SBT *χ^2^*_1,39_ = 3.65, p = 0.056). Tail chasers in BT (but not in other breeds) remembered frightening situations longer than the controls (Q 6d; BT *χ^2^*_1,52_ = 7.40, p = 0.007). The cases and the controls in none of the breeds differed in the amount of separation anxiety (results not shown).

We also found that tail chasers from SBT and BT (pooled data) were more keen on chasing moving objects (joggers, cyclers etc) (*χ^2^*_1,82_ = 4.07, p = 0.044) and possibly live prey (cats, birds etc.) (*χ^2^*_1,82_ = 3.21, p = 0.073). No differences were found in playfulness (*χ^2^*_1,82_ = 0.30, p = ns) or activity (*χ^2^*_1,82_ = 0.13, p = ns) compared to the control dogs. Analysing SBT and BT breeds separately did not yielded any significant associations, probably due to the low sample number.

Finally, we performed the analysis in the group including only the most severe cases (*TC_index_* 5–12) and the controls. We found similar results: tail chasers (BT, SBT, GS) (N = 24) were shyer (*χ^2^*_1,75_ = 6.39, p = 0.012), and less aggressive towards humans (*χ^2^*_1,75_ = 4.30, p = 0.038) compared to the controls (n = 51). Tail chasers (n = 24) in SBT and BT breeds also tended to be more interested in chasing moving objects (*χ^2^*_1,50_ = 3.10, p = 0.078) than the controls (n = 51).

### Candidate Locus Analysis

We established large pedigrees for each breed to evaluate the mode of inheritance of TC. The pedigrees indicate several affected individuals in many litters and suggest a strong genetic component, although it is unlikely to be a simple Mendelian disorder in any of the breeds (shortened pedigrees can be seen in **Figure S1**). The first locus for a canine compulsive behaviour was recently mapped to CFA7 for flank sucking behaviour [22]. We investigated whether the same locus is associated with TC by genotyping two SNPs around the CDH2 locus in 75 BTs, 23 SBTs and 47 GS (**Table 4**) and by sequencing the CDH2 coding regions in BTs. Both SNPs were polymorphic in all breeds but did not associate with TC in any of the studied breeds (**Table 4**). While sequencing the coding region we observed twelve polymorphisms by comparing the sequence of Bull Terriers to that of Boxer (sequence reference UniProtKB/TrEMBL: Q9GKK9 ). One SNP, c.1175G>A, in the coding region was observed to lead to the substitution p.Gly392Ser. Twenty-eight dogs, fifteen healthy dogs and thirteen affected, were then further genotyped for this SNP, however no correlation between the tail chasing status and the polymorphism was observed (results not shown).

**Table 4:** Candidate gene analysis of two SNPs in the candidate gene CDH2.

*Breed*	*SNP* a	*Case*	*Control*	*MAF*	*P*	*OR*
All breeds	1164			A(0.44)	0.886	
	**1144 (T/T)**	**58 (0.54)**	**49 (0.46)**	C(0.16)	**1**	**0.97**
BT^b^	1164			A(0.14)	0.283	
	**1144 (T/T)**	**40 (0.59)**	**28 (0.41)**	C(0.04)	**0.642**	**0.36**
GS	1164			C(0.22)	0.565	
	**1144 (T/T)**	**11 (0.41)**	**16 (0.59)**	C(0.29)	**1**	**0.96**
SBT	1164			G(0.33)	1	
	**1144 (T/T)**	**7 (0.58)**	**5 (0.42)**	C(0.35)	**0.667**	**0.54**

a Two SNPs were selected for analysis: 1) 1144 (63,867,472) as the T/T genotype for this SNP was previously associated with Doberman compulsive behaviour [22], and 2) 1164 (63,867,492) a nearby SNP which was included as it had more variation in all of the breeds. For 1144 the frequencies for genotype T/T (for cases and controls). minor allele frequencies (MAF) and odds ratio (OR) are presented. For 1164 minor allele frequencies (MAF) and the *P* value for SNP association is presented. All *P* values are calculated using Fisher’s Exact test.

b Bull Terrier includes both miniature and standard forms.

## Discussion

Several compulsive behaviours, often breed-specific, exist in dogs including fly-catching, flank sucking, freezing, chasing light reflections or shadows, spinning and tail chasing. Despite a suspected strong genetic component, the etiologies of these canine behaviours remain largely unknown and veterinary diagnoses have varied from opioid-mediated stereotypy to temporal lobe epilepsy and compulsive disorders [12], [13], [25]. Treatment trials on canine compulsive behaviours have reported partial responses to various therapies, i.e. anti-epileptics, anxiolytics, opioid antagonists, antidepressants and behaviour modifications [13], [17], [25], [28]–[31]. The first genetic evidence associated flank sucking in Dobermans with the *CDH2* locus [22], a gene which has also been associated with autism in humans. As repetitive behaviours are core features of autism, this finding supports the hypothesis that canine compulsive behaviours have common genetic antecedents with analogous behaviours seen in human neuropsychiatric disorders such as autism and OCD.

Utilizing detailed web-based questionnaires targeted to dog owners in several breeds, we characterized a classical canine compulsive behaviour, tail chasing, and potential associated environmental factors. Questionnaires have earlier been found to correspond well with actual behaviours of dogs and to provide valid methods for acquiring information on canine behaviour and personality [32]–[36]. For example, a recent questionnaire-based study of canine personality found a significant association between aggressiveness and an androgen receptor gene polymorphism in Akita Inus [37]. Owners often have a good picture of their dog’s behaviour (similar to parents living with their infants) and are usually pleased to participate in questionnaire surveys. Questionnaires have, in general, been found to have good test-retest reliability [38], [39]. Although the way that the owners interpret their dog’s feelings and emotions may vary, TC is a clearly recognisable behaviour and is easily describable for the owners.

We found a large variation in the frequency of tail chasing in each of the studied breeds, from zero to several times per day. Unlike the previous studies consisting mostly of clinical samples with more severe cases [25], [26], [40], our population sample covered the entire phenotypic range and included also milder cases in all breeds. The more severely affected dogs (*TC_index_* 5–12) represented only 23% of the cases in our study. This is in contrast to a recent TC study in BTs where a majority of the cases were recruited through a veterinary clinic resulting in a study population with of 74% severe cases with daily TC activities [26]. Different sampling strategies affect the composition of the study cohorts and need to be taken into account in the interpretation and comparison of findings.

We found an early onset of TC across breeds, varying from 3–6 months of age, thus preceding sexual maturity, as observed before in other canine compulsions [22], [26], [41]. In a recent large TC study in Bull Terriers, Moon-Fanelli et al. (2011) found males to be more susceptible to TC than females; however we did not observe any differences between males and females in tail chasing in any of the studied breeds [26]. Nor was there any difference between the males and females in TC frequency when only severe cases where included. The recent study found increased arousal/frustration to be the most frequent trigger among tail chasing Bull Terriers [26], whereas boredom was described as a trigger by one third of the owners in our study. This is in accordance with our finding that in households with more canine conspecifics or children (and therefore presumably more activity), dogs had less TC. Alternatively, the owners in these ‘busier’ homes (with several dogs or children) may not have had sufficient time to observe the dogs’ behaviour, causing a bias in the TC frequency. Tail chasers also seemed to be more interested in chasing moving objects (BT, SBT, GS) compared to dogs with no tail chasing. This may indicate a higher energy level and a possible lower threshold for frustration among tail chasers, but it may also represent an unusually strong drive to engage in a particular behaviour repertoire related to predation. In some cases TC behaviour may develop towards a reward seeking behaviour to reach the owner’s attention and consequently the dog may become conditioned to TC.

Almost half of our cases showed partially impaired responsiveness to owners’ calls during TC bouts, and this was clearly associated with the severity of TC. Previous studies on canine compulsive behaviours have reported a similar phenomenon [13], [24]. Impaired responsiveness during TC may be due to a reduced level of consciousness, a feature often associated with focal seizures. Although questionnaire data do not provide further resolution for this possibility, the seizure hypothesis for TC has been previously proposed based on clinical case studies showing a positive response to anti-epileptics and the presence of EEG abnormalities in TC dogs [25]. Although OCD and focal seizures can co-occur, human OCD patients typically do not show impaired responsiveness during the compulsive behaviour [42]. Alternative explanation for our finding of lowered responsiveness is that the compulsion might preoccupy the dog so strongly that it limits its apparent responsiveness even with a normal level of consciousness. In fact, earlier studies suggest a strong cognitive component for canine compulsive behaviours, where an animal restricted from performing compulsions may move out of the owner’s sight and continue the compulsive behaviour [13], [41]. Furthermore, 61% of the owners were able to stop TC by verbal commands, which would be unlikely in the case of an uncontrolled seizure episode.

Our TC dogs exhibited other stereotypic behaviours such as snapping at invisible flies or lights, trance-like freezing, licking and repetitive pacing. Similarly, tail chasing Bull Terriers were found to have more trance-like behaviour [26]. This group of investigators hypothesized that the common occurrence of trance-like “staring episodes” could support an association with autism spectrum disorders (in which such episodes can occur) or seizure activity. However, freezing or cessation of movement during performance of activities can also occur as part of the uncommon but well recognized manifestation of human OCD known as “obsessional slowness” [43]. As with most animal models, it is likely that TC and its associated phenomenon are better conceptualized as symptom clusters or “intermediate phenotypes” that are shared by multiple disorders (e.g. “repetitive behaviours” seen in both OCD and autism) rather than clinical human diagnoses [44]. Freezing was found predominantly in (n = 43) BTs, (both miniature and standard) and rarely in other breeds (n = 8), suggesting a strong breed-specificity. Breed-specific compulsions have been reported in several breeds [13], and although different compulsions may overlap and share biological etiology, it is also possible that they are genetically different, differ in the affected brain regions, or that certain behaviours become more predominant depending on the characteristics and functional purpose of the breeds. This would be analogous to human OCD, in which symptoms and co-morbidity varies significantly, leading to the consensus view that OCD is more of a spectrum of overlapping syndromes rather than a unitary disorder [1], [14].

### Diet Supplement, Neutering and Other Environmental Factors

TC was associated with dietary supplements, neutering status and also the number of conspesifics in the household. Furthermore, these factors were also associated with TC when comparing only severe cases to the controls. Our study revealed that dogs receiving dietary supplements chased their tails less compared to dogs not receiving any nutrients. This may be a correlative indication that dietary supplements lead to reduced TC. In addition, the data set including only tail chasers showed lower TC activity for dogs receiving dietary nutrients (in BT and SBT), further supporting an effect of dietary nutrients on TC behaviour.

Our rough categorization of the dietary supplements into five main groups, labelled vitamins, minerals, Omega 3, Omega 6 and ‘something else’ and comparison between cases and controls revealed that fewer tail chasers received vitamins and minerals, especially vitamin B6 compared to the control-dogs. Dogs receiving vitamins and minerals (especially vitamin B6 and C) also chased their tails less compared to the dogs not receiving these micronutrients. Interestingly, there are indications that vitamins and minerals have beneficial effect also in human OCD treatment. Two case studies report decreased anxiety and remission of OCD symptoms after treatment with EMPowerplus, which is a formula consisting of 14 vitamins, 16 minerals, 3 amino acids and 3 antioxidants [45], [46]. Although we are lacking in information on the actual frequency and quantity of the given supplements in our study, it is worth noting that only vitamin B6 showed a difference. Vitamins and minerals are known to be important for brain function [47]. Vitamin B6 is a common name for six different water-soluble compounds that are present in foods and in the body, of which pyridoxal phosphate (PLP) is the most common form (over 60% of the body’s vitamin B6 is PLP). All types of DNA and RNA syntheses are dependent on B6. In the brain, PLP-dependent enzymes are involved in the metabolism of many amino acid and amine neurotransmitters such as dopamine, serotonin, glutamate, glycine, GABA, D-serine and taurine [48]. They are also important in the synthesis of neuroprotective compounds such as kynurenic acid. Thus deficiency or defects in the metabolism of PLP would be expected to cause major neurological consequences [49]. Deficiency of vitamin B6 or a positive response to vitamin B6 therapy has long been known to be associated with neuropsychiatric illnesses such as depression in humans and epilepsy in children [50], [51]. Further, reduced levels of serotonin and increased dopamine, both dependent of vitamin B6, can both lead to OCD [52].

The minimum requirement of vitamin-B6 and Mg that a dog should get via its daily food is internationally agreed upon and should be 0.04 and 5.91 mg/kg bodyweight raised to 0.75, respectively [53]. This, however, might not be enough, especially if the dog has, a malfunctioning intestinal wall, a high stress level, an overweight diet, has too small food rations due to overweight, is on a long antibiotics or oestrogen medication, or has other components in its diet that hamper nutrient absorption etc. In this study we had detailed nutrient content information of only the supplements data and not of basic food. Therefore, we were not able to calculate the total nutrient ingestion per individual (including nutrient information of both food and diet supplements). Nevertheless, it would be interesting to give vitamin B6 and Mg supplement (and possible vitamins C, B5 and B12) to all TC dogs and see if any therapeutic help could be gained from them.

It is also possible that owners who give their dogs dietary supplements handle their pets differently in other respects that could also have a preventing effect on TC. However, our questionnaire covered a large spectrum of important issues of dog’s daily routines, which did not associate with either dietary supplements or TC. An earlier investigation about the role of dietary nutrients (zinc, copper and iron) on compulsive tail chasing in BT, however, did not find any association [54]. If vitamins and minerals have a real effect on TC, it could have important consequences for both the treatment and the possible etiology of compulsive behaviours.

We found that sterilized individuals, especially females, had less TC compared to intact dogs in a pooled analysis of all breeds. When only the more severe TC dogs were compared to the controls, neutering was significantly associated with TC in only females. Findings were somewhat inconsistent when analyzing within breeds, perhaps reflecting the smaller sample sizes for these analyses. In contrast to our study, a previous OCD study [41] found more neutered males among compulsive dogs (of all breeds) compared to other veterinary clinic patients. Their study, however, lacked proper breed controls, and included all forms of compulsions in the same analysis. In fact, gonadal steroids have been suggested to play a modulatory role in animal [55] and in human compulsions [56], where premenstruum, pregnancy and post-partum have increased risk of onset and deterioration of the OCD [55], [57]. Neutering in female dogs reduces the production of progesterone and oestradiol, which may have a controlling effect on compulsions. Most animal studies, however, have found that low levels of ovarian hormones worsen the symptoms [55], [58], whereas our results indicate the opposite in dog. Rat animal models differ from our canine model in many important aspects. Rat models are experimental and do not show spontaneous compulsive behaviour, as canine models do. The difference in the compulsive phenotypes may explain the differences in the response to hormonal fluctuation between the two models.

A recent study suggests that tail chasing in dogs might, at least partly, result from lack of activities, exercise or stimulation [24]. The research material in this study was 400 dog tail chasing videos collected from You Tube, with no other information on the dogs. The author based her conclusion on the fact that most tail chasing videos were recorded indoors, whereas ‘control’ videos (also collected from You Tube) were more often recorded outside. In our study, the owners reported in detail the daily exercise and activities of their dogs, and no association between tail chasing frequency and exercise or the amount of activities was found.

### Personality Differences

We found four major personality factors to exist based on the personality questionnaires; Shyness/Boldness, Sociability/Dogs (sociability towards dogs), Amicability/Humans (friendliness towards humans), and Aggressiveness/Humans (aggressiveness towards humans). Shyness/Boldness, Sociability and Aggressiveness personality factors have been also found earlier in dogs using both behavioural testing and questionnaires [34], [59], [60], and thus our findings provide further support for the existence of these personality traits in dogs.

Different personality types may confer differential susceptibility for compulsive behaviours, or alternatively compulsive behaviours may influence personality. We found that TC dogs were generally shyer (more fearful) and less aggressive towards humans (exhibited as barking, growling, or biting), and this was also true when only the extreme cases and controls were included. Tail chasers also exhibited more noise phobia, especially towards fireworks. Although we could not find an earlier study assessing the personality of compulsive dogs, one earlier study suggested an association between fearfulness and compulsive disorder [13]. Similarly, a recent Bull Terrier study [26] found tail chasers to have slightly more phobias compared with no tail chasers. Human OCD belongs to the group of anxiety disorders, which includes panic disorder, social phobia, specific phobia and generalized anxiety disorder [61]. Behavioural inhibition (BI), which is a human temperament style characterized by avoidance of novel stimuli (social and non-social), withdrawal and restraint, associates with certain types of OCD (checking, doubting, obsessing, neutralizing and hoarding) [62]. Shyness in dogs is described in similar terms to human BI and may represent the same personality trait. TC behaviour may also function as a coping strategy for fear [16], although fearful situations were rarely described as TC triggers in our study. Interestingly, a recent study reported lower 5-HT2A receptor bindings in the same corticol areas in both fearful and compulsive dogs, suggesting that these disorders may be interrelated [20], [63].

Early-life experiences may predispose to various types of anxiety. We found that TC dogs were separated earlier from their mothers and experienced lower quality care from their mothers (as assessed by their owners/breeders) compared to the control dogs. Our finding is in line with the hypothesis that early maternal separation increases the risk of stereotypic behaviours [64], however, this is the first time that this association has been observed in dogs. Childhood trauma and stressful events have been associated with OCD [6], suggesting that same environmental factors may influence the development of both dog and human compulsive behaviours.

A recent study suggested that tail chasing may relate to autism [26] and, in addition, the first gene to be associated with canine compulsive behaviour has also been linked to human autism [22]. Autism in humans affects child development in three main areas: language ability, social interaction and stereotypic behaviour [65]. Compulsive behaviour in our study was not associated with amicability or sociability towards humans or dogs, or aggressiveness towards dogs, which are relevant estimates of canine social interaction abilities. Tail chasers actually showed less aggressive behaviour towards humans compared to the control dogs. This dog-human aggressiveness, which is mainly territorial or fear-related aggressive behaviour towards strangers (i.e. not family members), differs from episodic aggressiveness, which means a recurrent, unprovoked attack towards humans, animals or objects. Moon-Fanelli et al. (2011) found episodic aggression to be more frequent among tail chasers in their BT study [26]. The Finnish population included only five dogs that have had episodic aggressiveness (four tail chasers, one control) and we feel that this data set is too small to draw any conclusions from.

### Genetic Analyses

TC is observed in some breeds more often than in others indicating that genetic factors play an important role. Although the definitive mode of inheritance is often difficult to estimate from pedigrees due to missing phenotypes, our pedigrees in all breeds suggest a strong genetic influence with multiple affected dogs across generations and even within several litters. No association was observed among the studied breeds between the TC and the CDH2 gene, which was previously found to associate with flank sucking in Dobermans [22]. We are currently conducting more extensive genetic analyses across breeds, which are likely to reveal novel loci for TC. Dietary supplements may suppress TC, and thus the use of supplements needs to be controlled in genetic case-control comparisons. We therefore re-checked the CDH2 candidate gene from the data, leaving out all control dogs receiving vitamins and minerals; however, again no association was found. Human OCD is genetically complex with several genes that confer susceptibility to specific components of OCD [14]. Dogs with compulsive behaviours may provide an alternative approach for elucidating the genetic basis of OCD and related disorders, since the unique population structure of domestic dogs has been demonstrated to provide an ideal model for mapping behavioural and medical traits [66], [67]. Furthermore, dog breeds provide genetic isolates, each having a specific predisposition towards compulsive behaviours, which increases the feasibility of conducting genetic association studies.

In summary, we describe here the first large-scale questionnaire study of TC in a pet population sample including four different dog breeds. Our study reveals several similarities between canine tail chasing and human OCD, together with a novel finding of the effect of dietary nutrients on TC. Altogether our results add more evidence for shared etiology and mechanisms of OCD in dogs and humans [6], and suggest that both environmental and genetic factors affect compulsive behaviour. Our pedigrees indicate a genetic contribution to tail chasing. We found no evidence for an association with a gene previously associated with canine compulsive behaviour, motivating our ongoing genetic analysis which hopefully will reveal novel loci and pathways for TC that can be replicated in human OCD cohorts.

## Materials and Methods

### Study Population

Behavioural data from Finnish Bull Terriers (Standard BT & Miniature MBT), German Shepherds (GS) and Staffordshire Bull Terriers (SBT) was collected using two owner-completed web-based questionnaires (**Attachment S1 and S2**). Questionnaires were sent to: 1) the owners who had already given blood samples to our Dog DNA bank, and 2) breed clubs (the Finnish Bull Terrier Association, the German Shepherd Union Finland and the Finnish Staffordshire Bull Terrier Association). The owners were invited to participate in the survey and asked to fill out the questionnaire whether their dog was a non-chaser or chaser. The first questionnaire (Stereotypic Behaviour Q) consisted of a series of questions including dogs’ a general history, environmental factors, diet, tail chasing history and the presence of other possible compulsive behaviours (**Attachment S1**). Altogether 368 questionnaires were received from four breeds (BT: 41 males and 56 females; MBT: 14 males and 11 females; GS: 93 males and 89 females; SBT: 32 males and 28 females). We also received few questionnaires (2 males and 3 females) from American Staffordshire Bull Terriers (AST), and because of the small number these were grouped together with SBT (**Table S1)**. Miniature and Standard breed variants of Bull Terriers were pooled in the later analysis into one group, named BT. Pooling of these two breeds was considered reasonable since these breeds are closely related (with shared breed standards) and are interbred in Europe and also in Finland.

As a measure of the frequency and intensity of tail chasing we used a variable *TC_index_*, which was based on the owner’s answers. *TC_index_* (0–12) is a sum of numerical answers to the questions “Does your dog chase its tail?” from ‘never’ to ‘multiple times per day’ and “On average, how much time in a normal day does the dog spend chasing its tail?” from 0 min up to 5 hours or more (**Attachment S1**). Dogs with no history of tail chasing were considered as controls (*TC_index_ = 0)*. In addition to using the variable *TC_index_* in the data analysis as a response variable, we also used a binary case-control set up (control =  no history of TC and case  = TC observed from “few time during the dog’s life” to “multiple times per day” (*TC_index_*≥*1*) in the analysis. Variables *amount of socialisation* (sum of all socialisation events reported) and *amount of exercise* (sum of the questions “How many times does your dog get exercise in a typical day” (scale 1–4) and *“*How many hours/minutes does your dog get exercise in a typical day*?*” (scale 1–5) were derived from the owners’ answers combining two or more related questions.

The second questionnaire, Dog’s Personality Q (**Attachment S2**) was sent to those SBT, BT and GS owners that had returned Stereotypic Behaviour Questionnaires and whose dogs had either *TC_index_*≥2 or *TC_index_* = 0. This questionnaire aimed to provide a personality profile of the dogs, especially their potential fearful or aggressive reactions to everyday situations (strange humans, novel situations, loud noises and separation from the owner), interest in play, and activity. The noise phobia –section was derived from a questionnaire developed for assessing the genetic basis of noise phobia [68] and some of the questions dealing with reactions to everyday situations were derived, with slight modification, from CBARQ [69]. In addition, we asked about the age of separation from the mother and the quality of maternal care on a scale of 1–6.

### Food and Dietary Supplement Data

As part of the Stereotypic Behaviour Questionnaire owners were also asked to report the type of food dogs received in five different categories: *homemade leftovers*, *homemade-especially for the dog, commercial (pet store), commercial (supermarket*) or *something else*. Since there was only one dog receiving *homemade leftovers* and nine dogs with *commercial (supermarket*), we regrouped the main categories by combining the original categories as follows: *homemade,* (both *homemade* categories) *something else*, *commercial* (both *commercial* categories) and special *raw food* diet (Bone And Raw Food). Owners were also asked if dogs received any dietary supplements. From the 231 dogs receiving dietary supplements, detailed nutritional supplement information was specified for 139 dogs. The nutrient content of the supplements was divided into five main groups; vitamins, minerals, Omega-3, Omega-6 and “something else”. The group “something else*”* included mainly different herbs, glucosamine and other joint products, some amino acids, polysaccharides or lactobacilli.

### Statistical Methods

The data collected via two questionnaires was analysed in two ways: all breeds pooled together and each breed separately. The possible association of TC with environmental factors in the pooled data and for each breed was further analysed with two methods. First, *TC_index_* was used as a response variable using a generalized linear model with either Poisson error or negative binomial structure in PROC GENMOD (SAS version 9.2) – in this analysis all individuals were included (*TC_index_* 0–12). Second, a case-control setup was used to compare differences between the most severe tail chasers (*TC_index_*5–12) and the controls (*TC_index_* = 0) using a generalized linear model with a binomial distribution (logistic regression). The following explanatory variables were included in the analysis: *gender, breed, age of arrival to a new home (in weeks), place of birth (at mother’s home/at breeder) amount of socialisation, number of children in the household, number of adults, number of dogs in the household, number of other diagnosed diseases, the time the dog has to spend alone during a normal day, amount of daily exercise, amount of activities done with the dog, dietary supplements, neutering status and type of food*. For most of the questions in the Stereotypic Behaviour Q we had answers from 258–367 dogs, except for the question ‘*time dog has to spend alone during normal day’* for which we received only 134 answers (question was added later to the Q). The Goodness of Fit value (describing how well the model fits into the distribution) was checked for each model and values close to 1.0 were considered a very good fit [70]. The best model was chosen based on both AIC criteria (Akaike’s Information Criteria, where the smaller the better) and Goodness of Fit value. Terms gender and breed and interactions were included for all models. Non-significant terms were dropped out individually and only the statistically significant terms were kept. Kruskall-Wallis nonparametric ANOVA and Spearman correlation were used in analyses that included only tail chasers (*TC_index_*≥2).

Personality data from BT, GS and from SBT (a total of 129 answers) was first analysed with factor analysis to assess latent variables and reveal possible inter-correlations between different variables in the questionnaire. All analysis was performed with SAS (version 9.2). Factor analysis (PROC FACTOR) was conducted using principal factor method with VARIMAX rotation. Questions from sections 1–7 were included in the factor analysis (noise phobia questions were combined as one value) (**Attachment S2**). Questions 9, 10 and 11 were left out from factor analysis due to the low number of answers (factor analysis does not handle missing values in SAS). Similarly, question 8 was left as it was in yes/no scale. Questions 9–11 were analysed in a separate analysis using a generalized linear model with a binomial error distribution. In factor analysis estimating the communalities we used squared multiple correlation (R^2^) (Option PRIORS = SMC). Two questions (7 and 6d) were excluded from the factor analysis due to low communality values, and these were also analysed separately using generalized linear model with binomial error distribution. Four factor models were chosen in both cases based on the inspection of the scree plot and residuals and partial correlations. The possible association of personality traits with TC behaviour was investigated using a generalized linear model with a binomial error distribution (PROC GENMOD) in SAS. In this model the status of the dog (case or control) was used as a binary response variable and the four personality factors as explanatory variables. In addition, explanatory variables included breed and gender and their interaction with other variables. The same statistical methods were also used in analysing the willingness to chase moving objects, chase living prey, playfulness and general activity. The possible association of personality with TC was analyzed for each breed separately as well as for all breeds together. Type 3 analysis statistics are presented, which means that the analysis is not dependent on the order in which the terms for the model are specified.

The effects of the mother’s quality of care, possible complications in birth, and age in weeks when separated from the mother were estimated using a generalized linear model with a binomial error structure in PROC GENMOD (SAS version 9.2). In the preliminary model we included tail chasing status (case/control) as binary response variable, and *mother’s quality of care*, *time of separation in weeks, birth (problems/no problems), breed, gender* as explanatory variables. As breed, gender and all the interaction terms were not statistically significant they were dropped out of the final model.

### Candidate Gene Analysis

In order to analyze the association of the Doberman flank sucking locus CDH2 we developed Taqman-based assays (Applied Biosystems) for two SNPs at positions 63.867.472 bp and 63.867.492 within the CDH2 gene, including the best associated SNP in the Doberman study (SNP 63,867,472) [22]. Altogether 80 (40 standard BTs, 4 mini BTs, 21 GS and 15 SBTs) cases (*TC_index_*≥2) and 66 (24 standard BTs, 4 mini BT, 30 GS and 8 SBTs) controls were genotyped. The study cohort included 12 Standard BT cases and 12 controls from France, while the rest of the samples originated from Finland. The French samples were recruited through a veterinary clinic and all the cases exhibited severe tail chasing, for which they needed veterinary consultation. A pedigree analysis of both the Finnish and the French dogs was used to ensure that the cases and the controls were unrelated at least at parental level. The association was analyzed by comparing the allele frequencies in the cases and the controls using the Fisher’s exact t-test. Additionally, coding regions and splice sites were sequenced according to Ensembl’s CDH2 gene reference Q9GKK9_CANFA ENSCAFG00000018115 from 4 (2 unrelated cases and 2 controls) French BTs, and from 2 (one case and one control) French MBTs. Primers are available upon request. Pedigrees were established with the help of the Finnish Breeding Association Registry.

### Ethics Statement

The blood sample collection from the privately-owned Finnish dogs was performed by trained professionals, with permission from the dog owners, and was conducted according to Finnish and international guidelines and laws. The French blood samples were collected in the veterinary clinic during veterinary consultation, with permission from the owners. We have a valid ethical license (Finnish National Animal Experiment Board, ELLA, license number ESLH-2009-07827/Ym-23, expiring Oct 2012). Dog behavior was assessed via questionnaires, and no behavioral experiments were conducted.

## Supporting Information

Figure S1 **Short examples of pedigrees of a) Bull Terrier b) Miniature Bull Terrier, c) German Shepherd d) Staffordshire Bull Terrier.** Dogs with *TC_inde_*_x_>2 are marked with black squares, dogs with mild TC (*TC_inde_*_x_ = 1) are marked with grey squares, and dogs marked with white squares are dogs with no TC or other stereotypy observed. Dogs inside a dotted line have some other stereotype than TC; (a) compulsive licking, d) compulsive drinking. The other dogs’ phenotype is unknown. Affected littermates had variation in *TC_inde_*_x_ in every breed. This is illustrated in one d) SBT family where all the dogs in the litter were affected – the number below the dog is *TC_inde_*_x_.
(TIF)

Table S1 **Demographic parameters of our study populations.**
(DOCX)

Movie S1 **Tail chasing in Bull Terrier puppy.** This female puppy is four months at the time of video shooting, and *TC_index_* is 9 (index varies between 0–12). Dog performs TC multiple times per day, and no clear reason for the behavior can be seen. Dog started the TC behavior at the age of 9 weeks.
(WMV)

Movie S2 **Tail chasing in German Shepherd male.** This is a four year old male at the time of video shooting and has *TC_index_* 9. Dog chases its tail multiple times per day and usually in situations where the dog has to wait for food or when owner talks to other people.
(WMV)

Movie S3 **Tail chasing in Staffordshire Bull Terrier male.** This dog is ten months of age, and all his sisters and brothers and a father (litter size 7) chase tails, while the mother does not have any stereotypic behavior. Dog has a *TC_index_* 7, and the owner reports that the dog may start TC when nobody pays attention to the dog. This dog also displays a trance-like behavior.
(WMV)

Attachment S1 **Stereotypic Behavior Questionnaire.**
(DOCX)

Attachment S2 **Dog Personality Questionnaire.**
(DOCX)
