# Supplementary figures and images for: Environmental Effects on Compulsive Tail Chasing in Dogs
Source: PLoS One. 2012 Jul 26;7(7):e41684. doi: 10.1371/journal.pone.0041684 (PMC3406045; doi:10.1371/journal.pone.0041684)

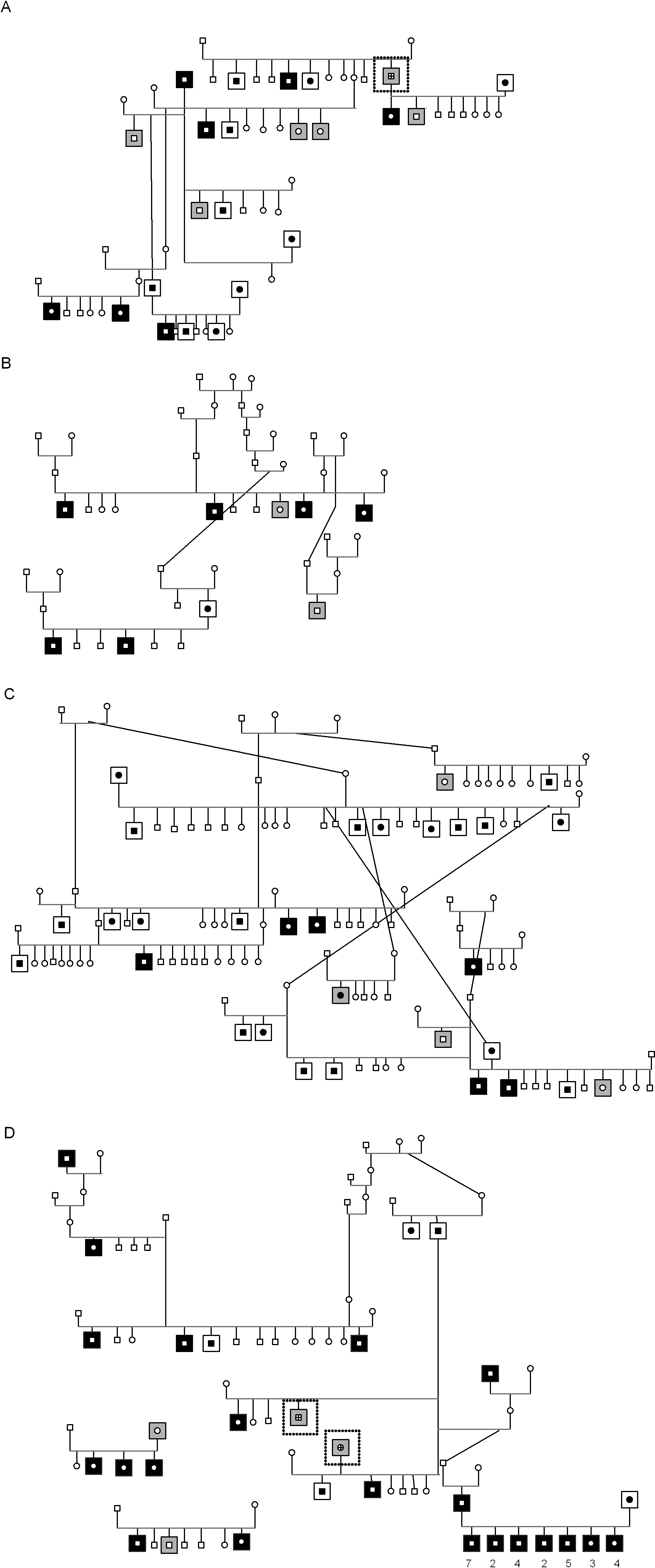

Supplement: Figure S1 — Short examples of pedigrees of a) Bull Terrier b) Miniature Bull Terrier, c) German Shepherd d) Staffordshire Bull Terrier. Dogs with TCinde x>2 are marked with black squares, dogs with mild TC (TCinde x = 1) are marked with grey squares, and dogs marked with white squares are dogs with no TC or other stereotypy observed. Dogs inside a dotted line have some other stereotype than TC; (a) compulsive licking, d) compulsive drinking. The other dogs’ phenotype is unknown. Affected littermates had variation in TCinde x in every breed. This is illustrated in one d) SBT family where all the dogs in the litter were affected – the number below the dog is TCinde x. (TIF) [file pone.0041684.s001.tif]
