# Supplementary material for: Environmental Effects on Compulsive Tail Chasing in Dogs
Source: PLoS One. 2012 Jul 26;7(7):e41684. doi: 10.1371/journal.pone.0041684 (PMC3406045; doi:10.1371/journal.pone.0041684)
Supplement: Table S1 — Demographic parameters of our study populations. (DOCX) [file pone.0041684.s002.docx]

**Table S1.** Demographic parameters of our study populations^a^.

| Breed | N | Median age (range) |
| --- | --- | --- |
| **Staffordshire Bull Terrier (60)** |  |  |
| Case | 36 | 4 (1.5-16) |
| Control | 24 | 6 (2-11) |
| **American Staffordshire Bull Terrier (5)** |  |  |
| Case | 1 | 1.5 |
| Control | 4 | 3.5 (1-12) |
| **German Shepherd (181)** | 38(17) |  |
| Case | 55 | 3 (1-10) |
| Control | 126 | 6 (1-12) |
| **Bull Terriers** |  |  |
| Standard (97) |  |  |
| Case | 65 | 2 (6m-8yrs) |
| Control | 32 | 4.5 (1-13) |
| Miniature (25) |  |  |
| Case | 18 | 2 (1-12) |
| Control | 7 | 2 (2-5) |
| **TOTAL (368)** |  |  |
| Case | 175 | 3 (6m-16 yrs) |
| Control | 193 | 4 (1-13yrs) |

^a^Stereotypic behaviour questionnaires were received from five breeds from a total 368 dogs. In the later analyses the five American Staffordshire Bull Terriers were grouped together with Staffordshire Bull Terrier due to small sample size, resulting in total four breeds. We also received answers from nine dead dogs (included in N total).
