# Supplementary material for: Environmental Effects on Compulsive Tail Chasing in Dogs
Source: PLoS One. 2012 Jul 26;7(7):e41684. doi: 10.1371/journal.pone.0041684 (PMC3406045; doi:10.1371/journal.pone.0041684)
Supplement: Attachment S1 — Stereotypic Behavior Questionnaire. (DOCX) [file pone.0041684.s006.docx]

#
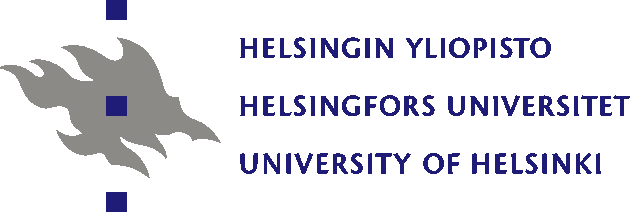
Stereotypic Behaviour Questionnaire

| Name of the owner: |
| --- |
| Address: |
| Phone number: |
| Email address: |
| Breed: |
| Dog’s name (official): |
| Registration number: |
| sex:  male  female |
| Is the dog spayed/neutered:  yes   no |

**Background information**

1. At what age did the dog enter your household?

2. Did the dog have any previous owners? Why didn’t the previous owners keep the dog?

3. Dog was acquired from  Home breeder

Home breeder (show dogs)

Home breeder (working dogs)

Large kennel

Larger kennel (show dogs)

Larger kennel (working dogs)

Other

4. Did the mother of your dog give birth to the litter at mother-dog’s owners home, or somewhere else e.g. in the breeder’s kennel)

In owners home

Not at home (e.g. In the breeder’s kennel)

I don’t know

5. When your dog was born, was there any complications etc. with the birth?

No

Yes, please specify

I don’t know

6. At what age was your dog separated from its mother? (By separation we mean the final physical separation from the mother – at latest this usually happens at the age of 7-10 week when the puppy arrives to a new home)

under 4 weeks

4 weeks

5 weeks

6 weeks

7 weeks

8 weeks

9 weeks

10 weeks

10-12 weeks

older than 12 weeks

the dog still lives together with its mother

7. How did the mother of your dog take care of the puppies?

I don’t know

Mother took excellent care of the puppies and spent a lot of time with them

Mother took care of the puppies reasonably well, but sometimes we had to command the mother to go to its puppies

At the beginning the mother spent time with the puppies, but later started to avoid spending time with its puppies

Mother did not want to spend time with the puppies, but still weaned enough, so that we did not have to get the puppies away from the mother

Mother did not want to spend time with the puppies, and we needed to bottle feed the puppies or use a ‘fostermother’.

How did the mother of your dog take care for the puppies? Something else

8. The socialization period: Did your dog experience the following events during the period between 7 weeks-3 months? How often?

*Events How often?*

Met strange adult dogs often sometimes never

Met strange children often sometimes never

Met strange men often sometimes never

Met strange women often sometimes never

Visited city (or other place with traffic &

many people) often sometimes never

Travelled by car often sometimes never

Travelled by train often sometimes never

Travelled by bus often sometimes never

9. Dog lives  indoors  outside/ in the kennel

partly inside/partly in the kennel/outside  other       (please specify)

10. What does your dog eat?

home food (specially made for the dog)

home food (leftovers)

commercial dog food (bought from pet shops)

commercial dog food (bought from the supermarket)

something else, please specify

11. Do you give your dogs extra vitamins/ dietary supplements?

Regularly  Sometimes  Never

If you give your dog extra vitamins / dietary supplements, please specify________________

12. How many times does your dog get exercise in a typical day?

three times or more

twice a day

once a day

dog is outside all the time

something else, please specify

During the daily walks, is your dog

on the leach during the whole walk

dog is leached part of the walk, and partly dog is allowed to run free

dog is allowed to run free during the walks

13. How many hours/minutes does your dog get exercise in a typical day?

three hours or more

2-3 hours

1-2 hours

30 min-1 hour

less than 30 min

14. Activities with the dog – please specify if you do any of these with your dog

obedience

tracking, searching, protection

volunteer rescue activity

dog shows

hunting tests

hunting with the dog

herding with the dog

agility

something else, please specify

15. Activities with the dog – how much do you spend time in activities mentioned above? (daily walking excluded)

zero

once a year

1-2 times / half a year

1-2 times / month

1-2 times / week

2-4 times / week

nearly daily

16. How much does your dog spend alone in the house/kennel during the average working day?

0 hours

0-1 hours

1-3 hours

3-6 hours

6-8 hours

8-9 hours

9-10 hours

10 hours or more

17. Your household includes       adults and       children

18. How many dogs do you have at the moment (please mention breed, sex and age of the dogs)

TAIL CHASING

19. Does your dog chase its tail?

Never observed

Rarely (Once or twice in lifetime)

Infrequently (less than once a month to once a year)

Frequently (less than once a week to once monthly)

Very frequently (every 2 days to once weekly)

Daily

Multiple times per day

On average, how much time on a normal day does the dog spend chasing its tail, or circling?

0 min

0-30 min

30 min – 1 hour

1-2 hours

2-3 hours

3-5 hours

5 hours or more

If your dog chases its tail, please specify a typical tail chasing situation:

On average, how long does one tail chasing bout last?

0-5 s

5-30 s

30 s – 1 min

1-5 min

5-10 min

10-15 min

15-30 min

30 min -1 hour

1-2 hours

2 hours or more

Fly snapping, chasing shadows, or lights

20. Does your dog try to catch ‘invisible flies’, or chase shadows or lights/reflections?

Never observed

Rarely (Once or twice in lifetime)

Infrequently (less than once a month to once a year)

Frequently (less than once a week to once monthly)

Very frequently (every 2 days to once weekly)

Daily

Multiple times per day

On average, how much time on a normal day does the dog spend in catching ‘invisible flies’?

0 min

0-30 min

30 min – 1 hour

1-2 hours

2-3 hours

3-5 hours

5 hours or more

On average, how long does one fly snapping bout last?

0-5 s

5-30 s

30 s – 1 min

1-5 min

5-10 min

10-15 min

15-30 min

30 min -1 hour

1-2 hours

2 hours or more

If your dog tries to snap invisible flies, chases shadows, lights or reflections, please specify a typical situation:

Licking, chewing, biting

21.Does your dog chew, bite or lick itself excessively, or licking surfaces, furniture’s etc?

Never observed

Rarely (Once or twice in lifetime)

Infrequently (less than once a month to once a year)

Frequently (less than once a week to once monthly)

Very frequently (every 2 days to once weekly)

Daily

Multiple times per day

On average, how much time on a normal day does the dog spend in licking, chewing or biting itself?

0 min

0-30 min

30 min – 1 hour

1-2 hours

2-3 hours

3-5 hours

5 hours or more

On average, how long does one licking bout last?

0-5 s

5-30 s

30 s – 1 min

1-5 min

5-10 min

10-15 min

15-30 min

30 min -1 hour

1-2 hours

2 hours or more

If your dog chews, bites or licks itself excessively, or licks surfaces, furniture’s etc, please specify a typical situation:

Has the chewing, biting or licking caused any self-injury to the dog (e.g.acral lick dermatitis)?  yes  no  I am not sure

Ramping, pacing

22. Does your dog exhibit “ramping” (excessive, repetitive walking or running such as running in “figure eight” pattern or along fences)?

Never observed

Rarely (Once or twice in lifetime)

Infrequently (less than once a month to once a year)

Frequently (less than once a week to once monthly)

Very frequently (every 2 days to once weekly)

Daily

Multiple times per day

On average, how much time on a normal day does the dog spend ramping?

0 min

0-30 min

30 min – 1 hour

1-2 hours

2-3 hours

3-5 hours

5 hours or more

On average, how long does one ramping bout last?

0-5 s

5-30 s

30 s – 1 min

1-5 min

5-10 min

10-15 min

15-30 min

30 min -1 hour

1-2 hours

2 hours or more

If your dog runs or walks in excess and in a repetitive manner, please specify a typical situation:

Freezing, trance-like behaviour

23. Does your dog exhibit slow walking, a trance-like behaviour where the dog typically walks in slow motion under curtains or tree leaves?

Never observed

Rarely (Once or twice in lifetime)

Infrequently (less than once a month to once a year)

Frequently (less than once a week to once monthly)

Very frequently (every 2 days to once weekly)

Daily

Multiple times per day

On average, how much time on a normal day exhibit trance-like behaviour?

0 min

0-30 min

30 min – 1 hour

1-2 hours

2-3 hours

3-5 hours

5 hours or more

On average, how long does one trance-like behaviour bout last?

0-5 s

5-30 s

30 s – 1 min

1-5 min

5-10 min

10-15 min

15-30 min

30 min -1 hour

1-2 hours

2 hours or more

If your dog exhibits freezing, trance-like behaviour, please specify a typical situation:

Fixated Staring

24. Does your dog have hallucinatory staring? (The dog might get ‘stuck’ in staring at a spot for a long time)

Never observed

Rarely (Once or twice in lifetime)

Infrequently (less than once a month to once a year)

Frequently (less than once a week to once monthly)

Very frequently (every 2 days to once weekly)

Daily

Multiple times per day

On average, how much time on a normal day does the dog spend hallucinatory staring?

0 min

0-30 min

30 min – 1 hour

1-2 hours

2-3 hours

3-5 hours

5 hours or more

If your dog exhibits fixated staring, please specify a typical situation:

Digging

25. Does your dog dig ground/floor excessively?

Never observed

Rarely (Once or twice in lifetime)

Infrequently (less than once a month to once a year)

Frequently (less than once a week to once monthly)

Very frequently (every 2 days to once weekly)

Daily

Multiple times per day

On average, how much time on a normal day does the dog spend digging?

0 min

0-30 min

30 min – 1 hour

1-2 hours

2-3 hours

3-5 hours

5 hours or more

On average, how long does one digging bout last?

0-5 s

5-30 s

30 s – 1 min

1-5 min

5-10 min

10-15 min

15-30 min

30 min -1 hour

1-2 hours

2 hours or more

If your dog digs excessively and in a repetitive manner, please specify a typical situation:

IF YOUR DOG EXHIBITS SOME OF THE ABOVE MENTIONED BEHAVIOURS, PLEASE ANSWER THE FOLLOWING QUESTIONS. PLEASE SPECIFY ON WHICH STEREOTYPIC BEHAVIOUR YOU ASNWER THE QUESTIONS (ie. tail chasing, licking, ramping, trance-like behaviour, staring or digging)?:

(IF YOUR DOG DOES NOT EXHIBIT ANY OF THE ABOVE MENTIONED BEHAVIOURS, PLEASE MOVE TO NEXT SECTION)

26. Does the stereotypic behaviour interfere with dog’s activities and usual functioning?

yes  no  I am not sure Please, specify

Have you ever tried to stop the behaviour?

yes  no  I don’t remember

27. If you answered YES to the previous question, does your dog stop the behaviour, when you tell it to stop?

yes, usually

yes, sometimes

yes, but the dog starts soon again

the dog does not stop the behaviour

28. Do you give medication for your dog because of the stereotypic behaviour? If yes, please specify the medication:

29. Is there any particular situation which usually precedes the stereotypic behaviour?

no, nothing particular

throwing a ball

playing, playfighting

stress

dog is bored, has too much energy

frightening situation

arrival of a guest

certain time of the day

something else, please specify

30. While behaving stereotypically, does the dog react to its name, or other commands?

yes, as usual

yes, but not as well as usual

not usually

no, never

31. If you dog behaves stereotypically, when did this behaviour start?

when the dog was between 3-6 months

when the dog was between 6 months and 1 year old

when the dog was between 1 and 1.5 years old

when the dog was between 1.5 and 2 years old

when the dog was between 2 and 3 years old

older, please specify

32. How does the tail chasing episode end? In what state is the dog? You can specify more than one option.

I can’t really say

normal, or nearly normal

tired

frightened

aggressive

absent

something else, please specify

33. Is there any physical injury to the dog’s tail due to tail chasing, licking etc?

yes no  I don’t know

34. Do you know if any of your dog’s relatives have tail chasing, or other unusual repetitive behaviour?

don’t know

does not have known relatives

Yes, the following relatives of dog have shown tail chasing or other unusual repetitive behaviours (indicate all that apply):

mother

please specify what stereotypic behaviour:

father

please specify what stereotypic behaviour:

sister

please specify what stereotypic behaviour:

brother

please specify what stereotypic behaviour:

grandmother

please specify what stereotypic behaviour:

grandfather

please specify what stereotypic behaviour:

daughter

please specify what stereotypic behaviour:

son

please specify what stereotypic behaviour:

other

Can you think of any other important information that might be related to your dog’s tail chasing behaviour?

DISEASES AND OTHER BEHAVIOUR

35. Does your dog have any of the following diseases:

Epilepsy  Diabetes

Allergies (e.g. food)  Demodex mites or demodicosis

Hypothyroidism  Glaucoma

Pancreatic insufficiency  Liver malfunction

Recurrent infections  Hereditary cataract

PRA (progressive retinal atrophy)  Malocclusion

Perianal fistula  Congenital heart failure

Cancer, what kind of?        Something else:

36. Has your dog ever exhibited fierce aggressive behaviour (episodic aggression), out of proportion to the situation in which it occurs or without any specific reason?

yes  no  I don’t know

If your answered YES to the previous question, please specify a typical situation in which episodic aggression occurs:
