# Supplementary material for: Environmental Effects on Compulsive Tail Chasing in Dogs
Source: PLoS One. 2012 Jul 26;7(7):e41684. doi: 10.1371/journal.pone.0041684 (PMC3406045; doi:10.1371/journal.pone.0041684)
Supplement: Attachment S2 — Dog Personality Questionnaire. (DOCX) [file pone.0041684.s007.docx]

# Dog Personality Questionnaire


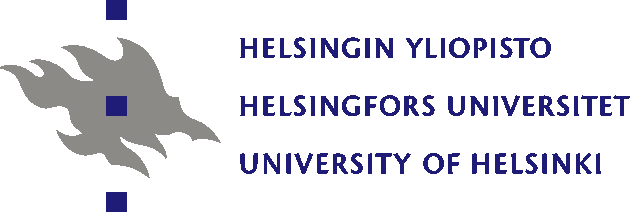


| Name of the owner: |
| --- |
| Address: |
| Phone number: |
| Email address: |
| Breed: |
| Dog’s name (official): |
| Registration number: |
| sex:  male  female |
| Is the dog spayed/neutered:  yes   no |

**Dog Personality Questionnaire**

With the help of the following questions, we try to build a clear image of the temperament and personality of Your dog. The following section includes questions of your dog’s behaviour in different situations. There are several questions from the same situations, focusing on the different areas of the behaviour (friendliness, fear, aggressiveness). When you think your dog’s behaviour, please answer according how the dog *usually behaves*, approx 50-100% of described events. In case none of the choices seem suitable, please describe the dog’s behaviour in an empty space. If your dog’s behaviour has changed, please describe the former and present behaviour.

1. FRIENDLINESS/ AMICABILITY. How does your dog usually (50-100% of the cases) behave when..
2. When an unknown ADULT tries to touch the dog while you are taking a walk

Dog is very eager and excited to get in contact with the stranger, overly friendly

Dog is very eager to get in contact with the stranger, but calms down easily

Dog is friendly, but calm, not excited

Dog is not interested to get in contact with the stranger, but is not afraid.

Dog is unwilling to get in contact with the stranger, backs down or tries to avoid physical contact

Dog behaves aggressively (barks, growls, or tries to bite)

Other

1. When an unknown CHILD tries to touch the dog while you are taking a walk

Dog is very eager and excited to get in contact with the stranger, overly friendly

Dog is very eager to get in contact with the stranger, but calms down easily

Dog is friendly, but calm, not excited

Dog is not interested to get in contact with the stranger, but is not afraid.

Dog is unwilling to get in contact with the stranger, backs down or tries to avoid physical contact

Dog behaves aggressively (barks, growls, or tries to bite)

Other

1. When a stranger tries to touch or stroke the dog at your home.

Dog is very eager and excited to get in contact with the stranger, overly friendly

Dog is very eager to get in contact with the stranger, but calms down easily

Dog is friendly, but calm, not excited

Dog is not interested to get in contact with the stranger, but is not afraid.

Dog is unwilling to get in contact with the stranger, backs down or tries to avoid physical contact

Dog behaves aggressively (barks, growls, or tries to bite)

Other

1. When the vet examines the dog

Dog is very eager and excited to get in contact with the stranger, overly friendly

Dog is very eager to get in contact with the stranger, but calms down easily

Dog is friendly, but calm, not excited

Dog is not interested to get in contact with the stranger, but is not afraid.

Dog is unwilling to get in contact with the stranger, backs down or tries to avoid physical contact

Dog behaves aggressively (barks, growls, or tries to bite)

Other

1. When a larger or same sized unfamiliar dog is approaching straight towards your dog.

Dog is very eager and excited to get in contact with the dog, overly friendly

Dog is very eager to get in contact with the dog, but calms down easily

Dog is friendly, but calm, not excited

Dog is not interested to get in contact with the dog, but is not afraid.

Dog is unwilling to get in contact with the dog, backs down or tries to avoid physical contact

Dog behaves aggressively (barks, growls, or tries to bite)

Other

1. When a smaller unfamiliar dog is approaching straight towards your dog.

Dog is very eager and excited to get in contact with the dog, overly friendly

Dog is very eager to get in contact with the dog, but calms down easily

Dog is friendly, but calm, not excited

Dog is not interested to get in contact with the dog, but is not afraid.

Dog is unwilling to get in contact with the dog, backs down or tries to avoid physical contact

Dog behaves aggressively (barks, growls, or tries to bite)

Other

1. When an unfamiliar dog is barking, growling or attacking towards your dog.

Dog is very eager and excited to get in contact with the dog, overly friendly

Dog is very eager to get in contact with the dog, but calms down easily

Dog is friendly, but calm, not excited

Dog is not interested to get in contact with the dog, but is not afraid.

Dog is unwilling to get in contact with the dog, backs down or tries to avoid physical contact

Dog behaves aggressively (barks, growls, or tries to bite)

Other

Changed behaviour

former

present:

1. FEAR. How does your dog usually (50-100% of the cases) behaves when..
2. When an unknown ADULT tries to touch the dog while you are taking a walk

No visible signs of fear (dog is either friendly on not interested)

Dog behaves aggressively (barks, growls)

Dog’s tail is between the legs, or in low position, takes contact carefully, is passive

Dog is unwilling to get in contact, might back down or try to avoid physical contact, but sometimes takes short contact (sniff)

Dog is unwilling to get in contact, backs down or tries to avoid physical contact, wants to get out of the situation

Other

1. When an unknown CHILD tries to touch the dog while you are taking a walk

No visible signs of fear (dog is either friendly on not interested)

Dog behaves aggressively (barks, growls)

Dog’s tail is between the legs, or in low position, carefully takes contact, is passive

Dog is unwilling to get in contact, might back down or try to avoid physical contact, but sometimes takes short contact (sniff)

Dog is unwilling to get in contact, backs down or tries to avoid physical contact, wants to get out of the situation

Other

1. When a stranger tries to touch or stroke the dog at your home

No visible signs of fear (dog is either friendly on not interested)

Dog behaves aggressively (barks, growls)

Dog’s tail is between the legs, or in low position, carefully takes contact, is passive

Dog is unwilling to get in contact, might back down or try to avoid physical contact, but sometimes takes short contact (sniff)

Dog is unwilling to get in contact, backs down or tries to avoid physical contact, wants to get out of the situation

Other

1. When the vet examines the dog

No visible signs of fear (dog is either friendly on not interested)

Dog behaves aggressively (barks, growls)

Dog’s tail is between the legs, or in low position, carefully takes contact, is passive

Dog is unwilling to get in contact, might back down or try to avoid physical contact, but sometimes takes short contact (sniff)

Dog is unwilling to get in contact, backs down or tries to avoid physical contact, wants to get out of the situation

Other

1. When a larger or same sized unfamiliar dog is approaching straight towards your dog.

No visible signs of fear (dog is either friendly on not interested)

Dog behaves aggressively (barks, growls)

Dog’s tail is between the legs, or in low position, carefully takes contact, is passive

Dog is unwilling to get in contact, might back down or try to avoid physical contact, but sometimes takes short contact (sniff)

Dog is unwilling to get in contact, backs down or tries to avoid physical contact, wants to get out of the situation

Other

1. When a smaller, unfamiliar dog is approaching straight towards your dog.

No visible signs of fear (dog is either friendly on not interested)

Dog behaves aggressively (barks, growls)

Dog’s tail is between the legs, or in low position, carefully takes contact, is passive

Dog is unwilling to get in contact, might back down or try to avoid physical contact, but sometimes takes short contact (sniff)

Dog is unwilling to get in contact, backs down or tries to avoid physical contact, wants to get out of the situation

Other

1. When an unfamiliar dog is barking, growling or attacking towards your dog.

No visible signs of fear (dog is either friendly on not interested)

Dog behaves aggressively (barks, growls)

Dog’s tail is between the legs, or in low position, carefully takes contact, is passive

Dog is unwilling to get in contact, might back down or try to avoid physical contact, but sometimes takes short contact (sniff)

Dog is unwilling to get in contact, backs down or tries to avoid physical contact, wants to get out of the situation

Other

Changed behaviour

former

present:

1. AGGRESSIVENESS. How does your dog usually (50-100% of the cases) behave when..
2. When an unknown ADULT tries to touch the dog while you are taking a walk

Dog *does not* bark, growl, or try to bite

Dog barks

Dog barks and /or growls

Dog barks and /or growls and tries to bite

Other

1. When an unknown CHILD tries to touch the dog while you are taking a walk

Dog *does not* bark, growl, or try to bite

Dog barks

Dog barks and /or growls

Dog barks and /or growls and tries to bite

Other

1. When a stranger tries to touch or stroke the dog at your home

Dog *does not* bark, growl, or try to bite

Dog barks

Dog barks and /or growls

Dog barks and /or growls and tries to bite

Other

1. When the vet examines the dog

Dog *does not* bark, growl, or try to bite

Dog barks

Dog barks and /or growls

Dog barks and /or growls and tries to bite

Other

1. When a larger or same sized unfamiliar dog is approaching straight towards your dog.

Dog *does not* bark, growl, or try to bite

Dog barks

Dog barks and /or growls

Dog barks and /or growls and tries to bite

Other

1. When a smaller, unfamiliar dog is approaching straight towards your dog.

Dog *does not* bark, growl, or try to bite

Dog barks

Dog barks and /or growls

Dog barks and /or growls and tries to bite

Other

1. When an unfamiliar dog is barking, growling or attacking towards your dog.

Dog *does not* bark, growl, or try to bite

Dog barks

Dog barks and /or growls

Dog barks and /or growls and tries to bite

Other

Changed behaviour

former

present:

1. SITUATIONS 1/FEAR . How does your dog usually (50-100% of the cases) behave when..
2. When you go to a dog show, or other big event with your dog

No visible signs of fear

Dog is unwilling to explore the environment; stays close the owner (without command)

Dog is unwilling to explore the environment, tail between the legs, or tail low, is passive

Dog is unwilling to explore the environment, tail between the legs, or tail low, is restless, might whine

Dog whines, pants, and tail is between the legs

Dog wants out of the situation, walks ‘low’, might pant, tail between the legs, or tail low

Other

1. When the dog faces new situations (first trip to the shopping mall, new house etc)

No visible signs of fear

Dog is unwilling to explore the environment, stays close the owner (without command)

Dog is unwilling to explore the environment, tail between the legs, or tail low, is passive

Dog is unwilling to explore the environment, tail between the legs, or tail low, is restless, might whine

Dog whines, pants, and tail is between the legs

Dog wants out of the situation, walks ‘low’, might pant, tail between the legs, or tail low

Other

1. In a heavy traffic

No visible signs of fear

Dog is unwilling to explore the environment, stays close the owner (without command)

Dog is unwilling to explore the environment, tail between the legs, or tail low, is passive

Dog is unwilling to explore the environment, tail between the legs, or tail low, is restless, might whine

Dog whines, pants, and tail is between the legs

Dog wants out of the situation, walks ‘low’, might pant, tail between the legs, or tail low

Other

Changed behaviour

former

present:

1. SITUATIONS 2/FEAR . How does your dog usually (50-100% of the cases) behave when..
2. When there is an unfamiliar object on the pathway of the dog ( or near the dog) like a plastic bag, leaves, flags waving etc.

Dog explores the object very quickly

Dog hesitates slightly, but explores the object relatively quickly

Dog hesitates slightly, might bark/growl, but goes usually without owner helping to explore the object

Dog hesitates, tail is low or between the legs, might bark/growl, explores the object only after owner goes first

Dog does not want to explore the object, wants out of the situation

Other

1. When the dog sees objects flown by the wind.

Dog explores the object very quickly

Dog hesitates slightly, but explores the object relatively quickly

Dog hesitates slightly, might bark/growl, but goes usually without owner helping to explore the object

Dog hesitates, tail is low or between the legs, might bark/growl, explores the object only after owner goes first

Dog does not want to explore the object, wants out of the situation

Other

1. Think how often the following sentence is true for your dog:
2. My dog is very eager to meet unknown people

never

seldom

sometimes

often

almost always

1. My dog is very eager to explore new places and situations

never

seldom

sometimes

often

almost always

1. My dog does not get easily frightened of strange objects or things.

never

seldom

sometimes

often

almost always

1. Its very difficult for my dog to get over from frightening situation, and it remembers it for a long time.

never

seldom

sometimes

often

almost always

Other things that are related to your dogs reactions towards unknown people, dogs, new

situations, and might be relevant for this study:

1. NOISE PHOBIA. Following questions deal with your dog’s reaction to loud noises. If your dog **reacts to loud noises, please mark yes, and choose the appropriate behaviour.** If your dog **does not react** on particular noise in question, mark **‘No’**.

If your dog’s behavior has changed at any time, please, describe the former and present behavior under ‘Changed behavior’.

THUNDERSTORM

YES  NO REACTION  DON’T KNOW

salivates

defecates

urinates

destroys

escapes

pants

hides

trembles

vocalizes

paces

freezes

tail low/ between legs

How often does the dog react to thunderstorm?

Always, 100% of the times

Almost always, 60-100% of the times

Often, 40-60% of the times

Rarely, 0-40% of the times

Changed behaviour

Former behaviour

Now

FIREWORKS

YES  NO REACTION  DON’T KNOW

salivates

defecates

urinates

destroys

escapes

pants

hides

trembles

vocalizes

paces

freezes

tail low/ between legs

How often does the dog react to fireworks?

Always, 100% of the times

Almost always, 60-100% of the times

Often, 40-60% of the times

Rarely, 0-40% of the times

Changed behaviour

Former behaviour

Now

GUNSHOTS

YES  NO REACTION  DON’T KNOW

salivates

defecates

urinates

destroys

escapes

pants

hides

trembles

vocalizes

paces

freezes

tail low/ between legs

How often does the dog react to gunshot?

Always, 100% of the times

Almost always, 60-100% of the times

Often, 40-60% of the times

Rarely, 0-40% of the times

Changed behaviour

Former behaviour

Now

OTHER NOISES (vacuum cleaners, leaf blowers, sirens, alarm systems etc.)

YES  NO REACTION  DON’T KNOW

salivates

defecates

urinates

destroys

escapes

pants

hides

trembles

vocalizes

paces

freezes

tail low/ between legs

How often does the dog react to gunshot?

Always, 100% of the times

Almost always, 60-100% of the times

Often, 40-60% of the times

Rarely, 0-40% of the times

Changed behaviour

Former behaviour

Now

How frequently do noise events such as thunder, fireworks or gunshots occur in the dog’s environment?

a.  Never

b.  Infrequently (few times a year)

c.  Regularly (averaging once a month or so)

d.  Frequently (a few times a month or more)

If you answered b, c or d what are the noises?

Has the dog ever been treated for noise sensitiveness or phobias? Please check all relevant choices below?

Acerpromazine

Rescue Remedy

Benzodiazepine, (Valiumilla or Xanaxilla

Other ’natural’ or ‘holistic’ remedies

Desensitization (tapes, CDs, videos)

Other?:

Do you have additional comments about your dog’s reaction to noise, or is there anything else about his or her behaviour when exposed to noise that you think we should know:

1. SEPARATION ANXIETY

Does your dog exhibit separation anxiety when left alone?

YES  / NO

If you answered yes, please explain how the dog behaves:

1. ACTIVITY

Please estimate your dogs general activity level

very calm, rather passive

calm

relatively active

active

very active

1. PLAYFULLNESS

How interested is your dog in playing with the owner/family member?

not interested

slightly interested

somewhat interested

interested

very interested

1. WILLINGNESS TO CHASE AFTER ESCAPING ANIMALS

How interested is your dog on chasing after escaping animal such as cat or a rabbit?

not interested

slightly interested

somewhat interested

interested

very interested
